# Multiscale Modeling and Dynamic Mutational Profiling of Binding Energetics and Immune Escape for Class I Antibodies with SARS-CoV-2 Spike Protein: Dissecting Mechanisms of High Resistance to Viral Escape Against Emerging Variants

**DOI:** 10.3390/v17081029

**Published:** 2025-07-23

**Authors:** Mohammed Alshahrani, Vedant Parikh, Brandon Foley, Gennady Verkhivker

**Affiliations:** 1 Keck Center for Science and Engineering, Graduate Program in Computational and Data Sciences, Schmid College of Science and Technology, Chapman University, Orange, CA 92866, USA; alshahrani@chapman.edu (M.A.); brfoley@chapman.edu (B.F.); 2 Department of Biomedical and Pharmaceutical Sciences, School of Pharmacy, Chapman University, Irvine, CA 92618, USA

**Keywords:** SARS-CoV-2 spike protein, Omicron variants, antibody binding, immune escape, molecular dynamics, protein stability, mutational scanning, binding energetics, evolutionary mechanisms

## Abstract

The rapid evolution of SARS-CoV-2 has underscored the need for a detailed understanding of antibody binding mechanisms to combat immune evasion by emerging variants. In this study, we investigated the interactions between Class I neutralizing antibodies—BD55-1205, BD-604, OMI-42, P5S-1H1, and P5S-2B10—and the receptor-binding domain (RBD) of the SARS-CoV-2 spike protein using multiscale modeling, which combined molecular simulations with the ensemble-based mutational scanning of the binding interfaces and binding free energy computations. A central theme emerging from this work is that the unique binding strength and resilience to immune escape of the BD55-1205 antibody are determined by leveraging a broad epitope footprint and distributed hotspot architecture, additionally supported by backbone-mediated specific interactions, which are less sensitive to amino acid substitutions and together enable exceptional tolerance to mutational escape. In contrast, BD-604 and OMI-42 exhibit localized binding modes with strong dependence on side-chain interactions, rendering them particularly vulnerable to escape mutations at K417N, L455M, F456L and A475V. Similarly, P5S-1H1 and P5S-2B10 display intermediate behavior—effective in some contexts but increasingly susceptible to antigenic drift due to narrower epitope coverage and concentrated hotspots. Our computational predictions show strong agreement with experimental deep mutational scanning data, validating the accuracy of the models and reinforcing the value of binding hotspot mapping in predicting antibody vulnerability. This work highlights that neutralization breadth and durability are not solely dictated by epitope location, but also by how binding energy is distributed across the interface. The results provide atomistic insight into mechanisms driving resilience to immune escape for broadly neutralizing antibodies targeting the ACE2 binding interface—which stems from cumulative effects of structural diversity in binding contacts, redundancy in interaction patterns and reduced vulnerability to mutation-prone positions.

## 1. Introduction

Structural and biochemical studies of the SARS-CoV-2 Spike (S) glycoprotein have revealed critical insights into the mechanisms driving viral transmission, immune evasion, and host–cell entry [1,2,3,4,5,6,7,8,9]. The S protein exhibits remarkable conformational flexibility, particularly within the S1 subunit, which includes the N-terminal domain (NTD), receptor-binding domain (RBD), and two structurally conserved subdomains, SD1 and SD2. This intrinsic flexibility enables the virus to dynamically adapt to different stages of infection, enhancing both its functional versatility and ability to evade immune detection [1,2,3,4,5,6,7,8,9]. The NTD facilitates initial attachment to host cells, while the RBD plays a central role in binding to the angiotensin-converting enzyme 2 (ACE2) receptor—a crucial step for viral entry [10,11,12,13,14,15]. Conformational changes between closed and open states allow the virus to efficiently engage with host receptors while leveraging structural variability to evade immune surveillance. This dynamic behavior not only enhances infectivity but also contributes to high transmissibility and pathogenicity by enabling immune escape [10,11,12,13,14,15]. Mutations within the S1 subunit can alter structural stability and conformational dynamics, influencing the toggling between open and closed states and modulating RBD accessibility—key determinants for viral attachment and immune recognition [16,17,18]. Cryo-electron microscopy (cryo-EM) and X-ray crystallography studies generated an extensive structural and functional atlas of the S protein in various functional states, including complexes formed with different classes of neutralizing antibodies [19,20,21,22,23,24,25].

The clade-based assignment of new Omicron variants is a critical framework for tracking the evolutionary trajectory of SARS-CoV-2 and understanding how ongoing mutations shape immune escape and viral transmissibility. This classification system, supported by Nextstrain, an open-source project for real-time tracking of evolving pathogen populations (https://nextstrain.org/ (accessed on 15 May 2025)), provides a phylogenetically grounded, standardized approach to interpreting viral evolution in real time. By identifying when a variant reaches at least 20% global prevalence and differs from its parent clade by at least two mutations, the clade-based model helps distinguish true antigenic shifts from background noise. The emergence and evolution of variants such as XBB.1 (clade 22F) and XBB.1.5 (clade 23A) reflect the virus’s adaptive capacity under selective pressures from immunity and transmission dynamics (Supporting Information, Figures S1–S3, Tables S1 and S2) [26,27,28]. XBB.1.5, a descendant of BA.2, carries RBD mutations that significantly enhance ACE2 binding affinity, increasing its infectivity compared to earlier Omicron strains [26,27,28]. Descendants like EG.5 and EG.5.1 further acquired the F456L mutation, improving immune escape [29,30,31,32]. Notably, “FLip” variants (e.g., JG.3, JF.1, GK.3, JD.1.1) independently evolved the L455F/F456L double mutation, illustrating convergent evolution driven by strong selective advantages that enhance immune evasion while maintaining receptor binding efficiency [33]. BA.2.86 exhibited significant genetic divergence and heightened immune evasion against RBD-targeted antibodies, surpassing even XBB.1.5 and EG.5.1 [34,35,36,37,38]. Its descendant, JN.1, acquired the L455S mutation, further enhancing immune escape at the cost of reduced ACE2 affinity [39]. Additional mutations at positions L455, F456, and R346 demonstrate the ongoing adaptive potential of the virus. The “SLip” variant combines L455S and F456L, allowing greater neutralization resistance [40,41,42,43], while the “FLiRT” variant adds R346T, maintaining ACE2 binding while improving immune evasion [40,41,42,43]. JN.1 subvariants KP.2 and KP.3 independently acquired mutations such as R346T, F456L, Q493E, and V1104L, enhancing transmissibility and immune evasion [44]. KP.3 (“FLuQE”) gained additional L455S mutations, conferring strong growth advantages. Other subvariants, including LB.1 and KP.2.3, share mutations like R346T and F456L while acquiring unique changes such as S31Δ and Q183H (LB.1) or H146Q (KP.2.3), contributing to increased immune evasion and higher reproduction numbers [45]. Among these, the F456L mutation plays a pivotal role in immune evasion, making KP.3 one of the most immune-evasive JN.1 lineages [46].

**Figure 1:**
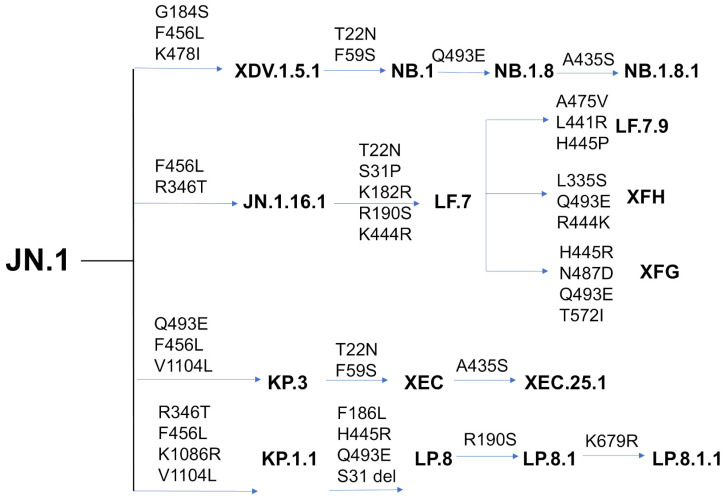
Phylogenetic Evolution of SARS-CoV-2 Variants Derived from JN.1 Lineage. Evolutionary trajectory of SARS-CoV-2 variants originating from the JN.1 lineage, highlighting key mutations that define distinct sublineages. The tree depicts the emergence of major variants such as XDV.1.5.1, NB.1.8, LF.7, KP.3, and their respective descendants, including NB.1.8.1, XFH, XFG, XEC.25.1. Key amino acid substitutions are annotated along each branch, indicating the genetic changes that drive variant divergence.

Recent cryo-EM studies show that the F456L mutation enhances Q493E-mediated ACE2 binding in KP.3, providing an evolutionary edge that allows immune-evasive mutations without compromising infectivity [47]. XEC, a recombinant variant derived from KP.3, gained attention due to NTD mutations F59S and T22N, increasing infectivity and immune resistance [48,49,50,51,52]. Structural analysis of KP.3.1.1 revealed epistatic interactions between F456L and Q493E: while Q493E alone reduces ACE2 binding, it synergizes with F456L to restore affinity [53,54]. Functional studies indicate that KP.3.1.1 exhibits reduced binding to monoclonal antibodies across multiple classes, partly due to S31Δ-introduced N30 glycosylation [53,54]. LP.8 carries notable mutations such as S31Δ, F186L, Q493E, and H445R, shaping its evolutionary trajectory. LP.8.1 (Clade 25A/Pango lineage) evolved into a “DeFLiRT” variant by losing S31 and acquiring Q493E and R190S [55] (Figure 1 and Figures S1–S3, Tables S1 and S2). While XEC and KP.3.1.1 dominate globally due to NTD mutations, emerging JN.1 sublineages like LF.7.2.1 and LP.8.1 demonstrate even greater growth advantages [53,54,55]. LF.7.2.1 features A475V, enhancing immune evasion over XEC [55], while LP.8.1 shows improved ACE2 engagement and humoral immune evasion, supporting its rapid spread [55] (Figure 1 and Figures S1–S3, Tables S1 and S2). A recent study identified BA.3.2, harboring over 50 mutations relative to ancestral BA.3, showing strong immune evasion, including resistance to Class 1/4 monoclonal antibodies, albeit with reduced ACE2 binding [56]. The latest variants NB.1.8.1, LF.7.9, XEC.25.1, XFH, and XFG displayed enhanced growth over LP.8.1.1, with NB.1.8.1 combining high ACE2 affinity and immune evasion through mutations G184S, F456L, K478I, T22N, F59S, Q493E, and A435S [56]. These findings highlighted the delicate balance between structural stability, immune evasion, and receptor binding that shapes the evolutionary trajectory of SARS-CoV-2 and its variants.

Antibody responses targeting the S protein are central to immunity. High-throughput yeast display screening and deep mutational scanning (DMS) have mapped functional epitopes targeted by neutralizing antibodies, enabling classification into distinct groups (A–F) [57,58]. Studies using DMS on XBB/JN.1 infections identified E1 group antibodies and F3 antibodies that exhibit broad neutralization capabilities [59,60,61]. Cao et al. demonstrated that aggregating DMS data and constructing pseudoviruses with predicted mutations significantly improves the identification of effective antibodies, increasing the success rate against XBB.1.5 from 1% to 40% [62]. Among them, BD55-1205 stands out as the only wild-type-elicited monoclonal antibody broadly potent against major variants, including XBB, BA.2.86, and JN.1-derived subvariants [62]. Using a yeast-display system combined with machine learning, a recent study identified VIR-7229, a class 1 antibody potently neutralizing EG.5, BA.2.86, and JN.1 [63]. Structural studies showed that VIR-7229 accommodates both F456 and L456 residues, tolerating high epitope variability with a high barrier to resistance [63].

Computer simulation studies provided important atomistic insights into understanding the dynamics of the SARS-CoV-2 S protein and binding with diverse binding partners. Molecular dynamics (MD) simulations of the full-length SARS-CoV-2 S glycoprotein with a complete glycosylation profile characterized the conformational landscapes of the S proteins in the physiological environments [64,65] and predicted the existence of multiple cryptic epitopes and hidden allosteric pockets [66]. Replica-exchange MD simulations explored dynamics of full-length S protein trimers, discovering transition pathways and previously unknown cryptic pockets that were consistent with FRET experiments [67,68]. Computational studies quantified the role of electrostatic interactions as a dominant thermodynamic force that differentiates binding strength of the Omicron S protein variants with the ACE2 receptor and antibodies [69]. Computational studies revealed distinct molecular strategies employed by broadly neutralizing antibodies: (1) conservation-driven binding, where antibodies exploit highly conserved residues critical for viral function, and (2) adaptability-driven binding, where antibodies utilize structural flexibility and compensatory interactions to tolerate mutations while maintaining neutralization efficacy [70,71,72]. At the molecular level, antigenic drift and convergent evolution shape immune escape hotspots through a fine-tuned balance of immune evasion and receptor binding, influenced by genetic mutations and antibody diversity. Functionally balanced substitutions that optimize tradeoffs between immune evasion, robust ACE2 affinity and conformational adaptability might be a common evolutionary strategy driving the emergence of new Omicron subvariants [73,74].

**Figure 2:**
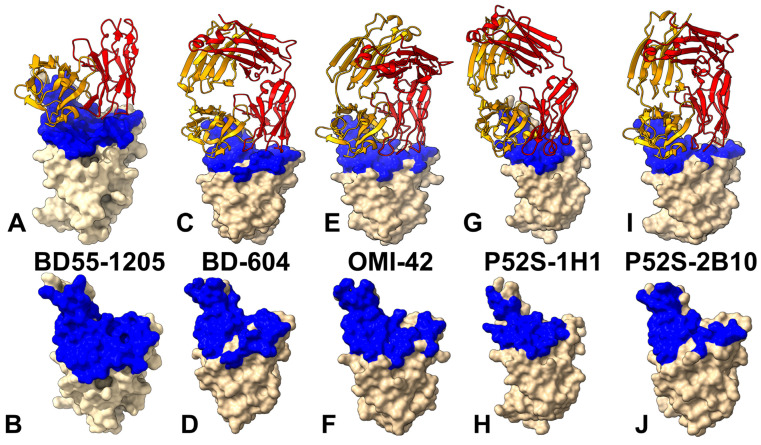
Structural organization of the RBD complexes and binding epitopes for class I antibodies. (**A**) BD55-1205 bound to XBB.1.5 RBD (PDB ID: 8XE9), with the heavy chain in orange and light chain in red. (**B**) Surface representation of the RBD highlighting the epitope footprint recognized by BD55-1205. The interface residues involved in antibody engagement are rendered in blue. (**C**) BD-604 complexed with RBD (PDB IDs: 7CHF, 7CH4), visualized using the same orientation as panel A. (**D**) Epitope localization for BD-604 on the RBD surface (blue), emphasizing its overlap with the ACE2-binding motif and shared contacts with BD55-1205. (**E**) OMI-42 in complex with Delta variant RBD (PDB ID: 8CBF), displayed with conserved orientation. Heavy and light chains are colored orange and red, respectively. (**F**) RBD surface view illustrating the epitope targeted by OMI-42, with interacting residues marked in blue. (**G**) P5S-1H1 bound to RBD (PDB ID: 7XS8). Antibody chains are represented in orange (heavy) and red (light) ribbons, with consistent orientation across the figure. (**H**) P5S-1H1 epitope mapped onto the RBD surface in blue, revealing partial overlap with ACE2 and distinct contact preferences compared to earlier panels. (**I**) P5S-2B10 bound to RBD (PDB ID: 7XSC), shown in the same orientation for comparative analysis. Orange and red ribbons denote heavy and light chains, respectively. (**J**) The P5S-2B10 epitope is displayed on the RBD surface (in blue), showing high similarity to P5S-1H1 but notable differences in hotspot residue involvement.

The interplay of dynamic and energetic factors is central to understanding how immune escape hotspots evolve, as these adaptations allow the virus to evade neutralizing antibodies without compromising its infectivity. In this study, we employed coarse-grained (CG) simulations using the CABS-flex approach that efficiently combines a high-resolution coarse-grained model and an efficient search protocol capable of accurately reproducing all-atom MD simulation trajectories and dynamic profiles of large biomolecules on a long time scale [75,76,77,78,79,80]. We performed multiple CG-CABS simulations of the S protein complexes with a panel of broadly neutralizing class I antibodies BD55-1205, BD-604, OMI-42, P5S-1H1 and P5S-2B10 (Figure 2), followed by all-atom reconstruction of trajectories to examine how structural plasticity of the RBD regions can be modulated by binding. Using dynamic ensembles of the antibody complexes and systematic mutational scanning of the RBD and antibody residues, we characterize patterns of mutational sensitivity and compute mutational scanning heatmaps to identify binding hotspot centers and characterize escape mutations. We also perform rigorous binding affinity computations of the antibody-RBD complexes and residue-based energy decomposition. Despite sharing similar epitopes and interaction footprints, we find that antibodies BD55-1205, BD-604, P5S-2B10, and P5S-1H1 can induce specific dynamic and energetically nuanced signatures. The results of this study underscore the fact that similar binding mechanisms for class I antibodies co-exist with subtle interaction differences that are influenced not only by the mutational variants but also by the characteristics of the antibodies. This antibody-dependent variability adds another layer of complexity to understanding how SARS-CoV-2 continues to adapt under selective pressures imposed by both natural immunity and vaccination.

## 2. Materials and Methods

### 2.1. Coarse-Grained Molecular Simulations and Atomistic Reconstruction of Equilibrium Ensembles

Coarse-grained (CG) models are computationally effective approaches for simulations of large systems over long timescales. We employed the CABS-flex approach that efficiently combines a high-resolution coarse-grained model and an efficient search protocol capable of accurately reproducing all-atom MD simulation trajectories and dynamic profiles of large biomolecules on a long-time scale [75,76,77,78,79,80]. In this high-resolution model, the amino acid residues are represented by Cα, Cβ, the center of mass of side chains and another pseudoatom placed in the center of the Cα-Cα pseudo-bond. In this model, the amino acid residues are represented by Cα, Cβ, the center of mass of side chains and the center of the Cα-Cα pseudo-bond. The CABS-flex approach implemented as a Python 2.7 object-oriented standalone package [75,76,77,78,79,80] was used in this study to allow for robust conformational sampling proven to accurately recapitulate all-atom MD simulation trajectories of proteins on a long time scale. Conformational sampling in the CABS-flex approach is conducted with the aid of Monte Carlo replica-exchange dynamics and involves local moves of individual amino acids in the protein structure and global moves of small fragments. The default settings were used in which soft native-like restraints are imposed only on pairs of residues fulfilling the following conditions: the distance between their *C*_α_ atoms was smaller than 8 Å, and both residues belong to the same secondary structure elements.

The crystal and cryo-EM structures of the Omicron antibody-RBD complexes are obtained from the Protein Data Bank [81] and the following systems were used in this study: the crystal structure of BD55-1205 with XBB.1.5 RBD (pdb id 8XE9), class I antibodies OMI-42 with Delta RBD (pdb id 8CBF), OMI-42 bound with Beta RBD (pdb id 7ZR7), BD-604 bound with RBD (pdb id 7CHF, 7CH4), BD-604 bound with BA.2 RBD (pdb id 8HWT) BD-604 bound with BA.4 RBD (8HWS), P5S-1H1 bound with RBD (pdb id 7XS8), and P5S-2B10 bound with RBD (pdb id 7XSC). A total of 100 independent CG-CABS simulations were performed for each of the systems studied. In each simulation, the total number of cycles was set to 10,000 and the number of cycles between trajectory frames was 100. MODELLER-based reconstruction of simulation trajectories to all-atom representation provided by the CABS-flex package was employed to produce atomistic models of the equilibrium ensembles for studied systems [82]. The CG conformational ensembles of the antibody-RBD complexes were also subjected to all-atom reconstruction using the PULCHRA method [83] and CG2AA tool [84]. The all-atom conformations were additionally optimized using the 3Drefine method [85] that utilizes atomic-level energy minimization with composite physics and knowledge-based force fields.

### 2.2. Binding Free Energy Computations: Mutational Scanning Profiling and Analysis

We conducted mutational scanning analysis of the binding epitope residues for the S RBD-antibody complexes. Each binding epitope residue was systematically mutated using all substitutions and corresponding protein stability and binding free energy changes were computed. BeAtMuSiC approach [84,85,86,87,88] was employed, which is based on statistical potentials describing the pairwise inter-residue distances, backbone torsion angles and solvent accessibilities, and considers the effect of the mutation on the strength of the interactions at the interface and on the overall stability of the complex. The approach evaluates the impact of mutations on both the strength of interactions at the protein-protein interface and the overall stability of the complex using statistical energy functions for ΔΔ*G* estimation, derived from the Boltzmann law, which relates the frequency of occurrence of a structural pattern to its free energy. BeAtMuSiC identifies a residue as part of the protein-protein interface if its solvent accessibility in the complex is at least 5% lower than its solvent accessibility in the individual protein partner(s). The binding free energy of protein-protein complex can be expressed as the difference in the folding free energy of the complex and folding free energies of the two protein binding partners:

(1) $$\Delta G_{bind}=G^{com}-G^{A}-G^{B}$$

(2) $$\Delta \Delta G_{bind}=\Delta G_{bind}^{mut}-\Delta G_{bind}^{wt}$$

We leveraged rapid calculations based on statistical potentials to compute the ensemble-averaged binding free energy changes using equilibrium samples from simulation trajectories. The binding free energy changes were obtained by averaging the results of over 1000 equilibrium samples for each of the systems studied. The ensembles are obtained using all-atom reconstructed trajectories obtained from CG-CABS simulations.

### 2.3. Binding Free Energy Computations

We calculated the ensemble-averaged changes in binding free energy using 1000 equilibrium samples obtained from simulation trajectories for each system under study. MM-GBSA binding free energy calculations were performed using all-atom models, which were reconstructed from CG simulations using the PULCHRA [83] and CG2AA [84] tools. CG-CABS were employed primarily for conformational sampling while the final energetic decomposition—including per-residue contributions and mutational scanning—was based on high-resolution all-atom conformations, ensuring accurate evaluation of van der Waals, electrostatic, and solvation effects.

Initially, the binding free energies of the RBD-antibody complexes were assessed using the molecular mechanics generalized Born surface area (MM-GBSA) approach [89,90]. Additionally, we conducted an energy decomposition analysis to evaluate the contribution of each amino acid during the binding of RBD to antibodies [91,92]. The binding free energy for the RBD-Antibody complex was obtained using:

(3) $$\Delta G_{\mathrm{bind}}=G_{\mathrm{RBD}-\mathrm{AB}}-G_{\mathrm{RBD}}-G_{\mathrm{AB}}$$

(4) $$\Delta G_{bind,MMGBSA}=\Delta E_{MM}+\Delta G_{sol}-T\Delta S$$

where ΔE_MM_ is total gas phase energy (sum of ΔEinternal, ΔEelectrostatic, and ΔEvdw); ΔGsol is sum of polar (ΔGGB) and non-polar (ΔGSA) contributions to solvation. Here, G_RBD–ANTIBODY_ represents the average over the snapshots of a single trajectory of the complex. G_RBD_ and G_ANTIBODY_ correspond to the free energy of RBD and antibody, respectively.

The polar and non-polar contributions to the solvation free energy are calculated using a Generalized Born solvent model and consideration of the solvent accessible surface area [93]. MM-GBSA is employed to predict the binding free energy and decompose the free energy contributions to the binding free energy of a protein–protein complex on a per-residue basis. The binding free energy with MM-GBSA was computed by averaging the results of computations over 1000 samples from the equilibrium ensembles.

In this study, we choose the “single-trajectory” protocol (one trajectory of the complex) to reduce the noise in the calculations due to the cancelation of intermolecular energy contributions. Entropy calculations typically dominate the computational cost of the MM-GBSA estimates. Therefore, it may be calculated only for a subset of the snapshots, or this term can be omitted [94,95]. In this study, the entropy contribution was not included in the calculations of binding free energies of the RBD-antibody complexes because the entropic differences in estimates of relative binding affinities are expected to be small owing to small mutational changes and preservation of the conformational dynamics [94,95]. MM-GBSA energies were evaluated with the MMPBSA.py script in the AmberTools21 package [96] and gmx_MMPBSA, a new tool to perform end-state free energy calculations from CHARMM and GROMACS trajectories [97].

## 3. Results

### 3.1. Structural Analysis of the RBD Complexes

Structural mapping of the RBD–antibody complexes for Class I neutralizing antibodies BD55-1205, BD-604, OMI-42, P5S-1H1, and P5S-2B10 reveals both shared features and functionally relevant distinctions in their epitope organization (Figure 2, Supporting Information, Tables S3–S7). The interfacial contacts and various types of intermolecular interactions (charged-charged, polar-polar, polar-nonpolar, nonpolar-nonpolar, charged-polar, and charged-nonpolar) are evaluated using PRODIGY approach in which the contacts are formed between amino acids at the interface of a protein complex within a specific distance threshold of 5.5 Å [98]. These antibodies target the apical head of the RBD, a highly conserved region critical for ACE2 engagement, ensuring effective blocking of viral entry. However, their epitope coverage, hotspot localization and reliance on specific residues combined with interaction diversity contribute to variations in neutralization breadth, escape resistance, and potency. A detailed comparison of the epitope footprints shows that BD55-1205 and BD-604 engage an extensive network of residues including Y421, Y453, L455, F456, Y473, A475, G476, N487, Y489, Q493, and H505, many of which are part of the receptor-binding motif (RBM) and contribute to both hydrophobic core stability and specific electrostatic interactions with the antibody (Figure 2A–D, Supporting Information, Tables S3 and S4). BD55-1205, in particular, spans over 20 residues, forming a continuous epitope across the RBD ridge, with key segments including 454–460, 486–494, and 498–505, which are central to both ACE2 recognition and antibody binding (Figure 2A,B, Supporting Information, Table S3). Among this panel of class I neutralizing antibodies, BD55-1205 engages the largest number of RBD residues, particularly in the RBM region. It is well-established that many RBM residues involved in ACE2 binding are also frequently mutated in Omicron variants including L455, F456, A475, F486, N487, F490, Q493, G496, Q498, N501, and Y505. All these residues participate in multiple interaction contacts with BD55-1205 and form the basis for a large broadly distributed binding epitope (Figure 2A,B, Supporting Information, Table S3).

BD-604 targets a similar but somewhat less extensive epitope (including residues Y421, Y453, L455, F456, Y473, A475, G476, N487, Y489, Q493, and H505) [98,99] but relying more heavily on side-chain contacts rather than backbone-mediated hydrogen bonds, which could limit its adaptability to mutational changes in the RBD (Figure 2C,D, Supporting Information, Table S4). OMI-42 shares many epitope residues with BD55-1205 and BD-604, particularly within segments 453–460 and 473–477, which participate in favorable contacts at the receptor-binding ridge [100,101] (Figure 2E,F, Supporting Information, Table S5). However, OMI-42 engages fewer RBM residues in the 486–494 region and lacks significant interactions with F486, F490, L492, S494, and G496 (Supporting Information, Table S5).

In contrast, OMI-42 (Figure 2E,F), P5S-1H1 (Figure 2G,H), and P5S-2B10 (Figure 2I,J) display more localized epitopes, with reduced coverage of the RBM and a greater reliance on side-chain interactions that are more sensitive to single-point mutations. These structural variations highlight that even closely related antibodies can adopt functionally distinct modes of engagement that could underpin their neutralization breadth, adaptability to mutations, and competition with ACE2 for receptor binding (Figure 2, Supporting Information, Tables S3–S7). The hydrogen bonding is formed by RBD residues R403, N405, T415, N417, D420, N487, Y489, Q493, Y501 and H505 interacting with Y33, S56, R97, R102 and E104 on the heavy chain and N30, D93 on the light chain of the BD55-1205 antibody [62] (Figure 2A,B, Supporting Information, Table S3). Strikingly, hydrogen bonds are formed between the backbone atoms of nine RBD residues—L455, R457, K458, Q474, A475, G476, S490, L492, and G502—and complementary residues in the heavy chain including T28, R31, N32, Y33, P53, and R102 [62] (Figure 2A,B; Supporting Information, Table S3). This extensive network of hydrogen-bonded interactions combined with strong hydrophobic contributions from residues Y453, L455, and F456 produces a highly stable and resilient binding interface. Importantly, the network of backbone-mediated hydrogen bonds with the RBD residues can enhance adaptability of BD55-1205 to mutations outside the primary epitope [62].

P5S-2B10 and P5S-1H1 exhibit a nearly identical binding pose to the top face of the RBD, mimicking the binding mode of ACE2 [102]. The binding epitope residues for P5S-1H1 is formed by residues R403, T415, G416, K417, D420, Y421, Y453, L455, F456, R457, K458, N460, Y473, Q474, A475, G476, F486, N487, Y489, Q493, Y495, Q496, Q498, T500, N501, G502, V503 and Y505 (Figure 2G,H, Supporting Information, Table S6). As a result, P5S-1H1 shares considerable similarities with BD-604 and BD55-1205, targeting residues within the hydrophobic core and mimicking interactions with the RBM region, including residues 473–477, 484–490, 493–505. The binding epitope residues for P5S-2B10 consists of residues R403, T415, G416, K417, D420, Y421, Y453, L455, F456, R457, K458, N460, Y473, A475, G476, F486, N487, Y48, Q493, N501, G502 and Y505 (Figure 2I,J, Supporting Information, Table S7). Both P5S-1H1 and P5S-2B10 emphasize residues Y421, Y453, and Y473, which are part of the hydrophobic core and critical for stabilizing the interaction. The stretch of residues 453–460 forms a hydrophobic core critical for binding stability of all examined class I antibodies. Among these residues, L455 and F456 are particularly important, as these positions are convergent evolution hotspots mutated in many recent variants. Structural analysis suggested that backbone-mediated contacts of these RBD residues with BD55-1205 may contribute to the unique binding and immune resistance profile of this antibody.

### 3.2. Coarse-Grained Simulations and Atomistic Reconstruction of the Conformational Ensembles for RBD Complexes with Class I Antibodies

**Figure 3:**
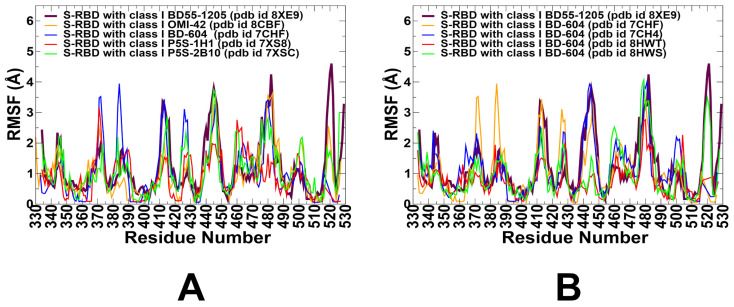
Comparative RMSF analysis of RBD dynamics upon binding by class I antibodies. (**A**). The RMSF profiles for the RBD residues obtained from simulations of the S-RBD complexes with class I antibodies: BD55-1205 with XBB.1.5 RBD, pdb id 8XE9 (in maroon thick lines), OMI-42 with Delta RBD, pdb id 8CBF (in orange lines), BD-604 with RBD, pdb id 7CHF/7CH4 (in blue lines), P5S-1H1 with RBD, pdb id 7XS8 (in red lines), and P5S-2B10 with RBD, pdb id 7XSC (in green lines). (**B**). The RMSF profiles for the RBD residues obtained from simulations of RBD with BD55-1205 (in maroon lines) and different structures of BD-604 antibody with the S-RBD complexes: BD-604 bound with RBD, pdb id 7CHF (in orange lines), BD-604 bound with RBD, pdb id 7CH4 (in blue lines), BD-604 bound with BA.2 RBD, pdb id 8HWT (in red lines), BD-604 bound with BA.4 RBD, pdb id 8HWS (in green lines). Conformational dynamics RMSF profiles show residue-level flexibility in the RBD when bound to BD55-1205 (8XE9, maroon), OMI-42 (8CBF, orange), BD-604 (7CHF/7CH4, blue), P5S-1H1 (7XS8, red), and P5S-2B10 (7XSC, green). Structurally conserved regions exhibit low fluctuations, while loop regions—especially those within the receptor-binding motif—display increased mobility depending on antibody engagement.

We performed multiple CG-CABS simulations of the SARS-CoV-2 S RBD-antibody complexes followed by all-atom reconstruction of trajectories to examine how structural plasticity of the RBD regions can be modulated by binding of antibodies. The root-mean-square fluctuation (RMSF) profiles provide a detailed view of the dynamic behavior of RBD residues upon antibody binding, highlighting both shared features and notable differences among the antibodies. The primary objective of this study was to investigate the dynamic and energetic contributions of RBD residues, as these residues play a pivotal role in mediating interactions with neutralizing antibodies. The RMSF profiles revealed generally similar dynamic behavior for RBD residues across all antibodies, reflecting the conserved structural core of the RBD. The central β-sheet and α-helices of the RBD (residues 350–360, 375–380, 394–403) exhibit low RMSF values, indicating minimal flexibility across all complexes (Figure 3). These regions are critical for maintaining the overall structural integrity of the RBD.

BD55-1205 induces significant stabilization in RBD regions such as 420–435, 450–475, and 490–505, where residues experience markedly smaller fluctuations compared to other antibodies (Figure 3A). This immobilization locks the RBD into a rigid conformation, reducing ACE2 engagement and enhancing neutralization efficacy (Supporting Information, Figure S4A). We simulated several structures of BD-604 complexes to obtain a more diverse assessment of the RBD mobility induced by this antibody (Figure 3B and Figure S4B). The RMSF profiles suggested that BD-604 can also cause significant stabilization of the RBD, including regions 375–390. The interfacial RBD positions involved in contacts with BD-604 (residues 490–505) also exhibit reduced flexibility, consistent with their role in stabilizing the antibody-RBD interactions (Figure 3B). The results also revealed important differences, particularly highlighting the subtle specifics of BD55-1205 and BD-604 dynamics. BD-604 binding can enable somewhat larger fluctuations in residues 450–475, suggesting that despite similar binding epitopes and interaction footprints, these class I antibodies can induce specific dynamic signatures. This is consistent with BD-604 exhibiting adaptability to substitutions at specific positions in its complementarity-determining regions (CDRs), such that interact with RBD residues Y473, Q474, A475, G476 [99]. Importantly, the results suggest that BD55-1205 binding can cause the larger stabilization and immobilization of the RBD interface, which can manifest in the increased RBD stability and stronger binding interactions.

For OMI-42, we found greater stabilization in regions 380–400 and 450–475. This stabilization likely reduces the flexibility of the 470–490 loop, enhancing its ability to block ACE2 binding (Figure 3A and Figure S4C). P5S-2B10 and P5S-1H1 exhibit intermediate behavior between BD55-1205 and BD-604 as these antibodies do not induce the same level of immobilization as BD55-1205 (Figure 3A and Figure S4D,E). Regions such as 490–505 show moderate fluctuations, indicating partial stabilization of the RBD interface. Intermediate stabilization suggests that P5S-2B10 and P5S-1H1 maintain a balance between flexibility and rigidity, allowing them to adapt to certain mutations while retaining binding efficacy (Figure 3A). The 470–490 loop of the RBM is a highly dynamic region of the RBD, located near the ACE2-binding interface and implicated in stabilizing the RBD-ACE2 interaction. Conformational ensembles obtained from simulations of all complexes (Supporting Information, Figure S4) suggested that the RBM regions exhibited elevated flexibility, though subtle differences were observed depending on the antibody. BD55-1205 can induce stabilization of 490–505 and indirectly restricts the flexibility of the 470–490 loop, locking the RBD into a rigid conformation. At the same time, BD-604 may induce more flexibility in the RBD residues 375–390 and 400–420. Combined with the greater mobility in the 470–490 loop, these dynamic signatures of BD-604 binding could make it potentially less effective in blocking ACE2 interactions (Figure 3A).

To summarize, the results highlighted subtle but important distinctions in the dynamic signatures of the RBD induced by class I antibodies. The observed dynamic modulation of the RBD by class I antibodies reflects a spectrum of binding strategies—from rigidification and global stabilization (BD55-1205) to localized flexibility and intermediate rigidity (P5S-1H1 and P5S-2B10), and to a more flexible and adaptable interface (BD-604) that is functionally effective but may be compromised in latest Omicron subvariants. These findings underscore that neutralization mechanisms are shaped not only by static epitope mapping and direct interaction contacts, but also by how each antibody modulates and reshapes the RBD conformational landscape, influencing its structural plasticity and mutational tolerance.

### 3.3. Mutational Profiling of Antibody-RBD Binding Interactions Interfaces Reveals Molecular Determinants of Immune Sensitivity and Emergence of Convergent Escape Hotspots

**Figure 4:**
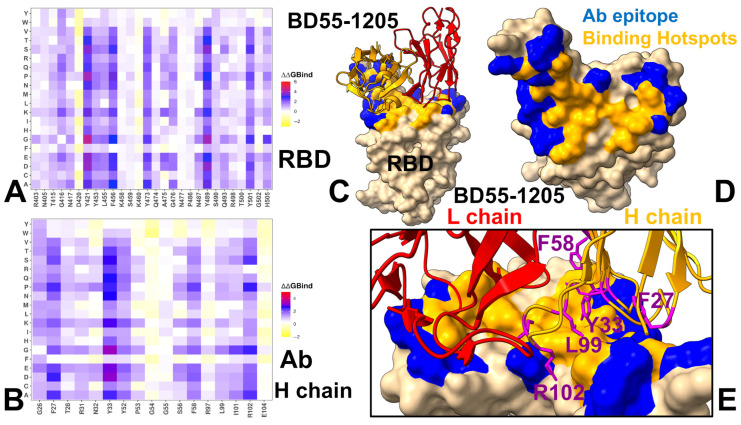
Mutational scanning of BD55-1205–RBD binding interface. (**A**) Mutational heatmap for interfacial RBD residues reveals key positions contributing to BD55-1205 binding stability. Destabilizing mutations (positive ΔΔG values) highlight escape-prone sites such as L455, F456, A475, Y489, and H505. (**B**) Complementary mutational profiling of the antibody heavy chain residues identifies F27, Y33, F58, L99, and R102 as critical for maintaining high-affinity binding. (**C**) Structural view of the BD55-1205–RBD complex (PDB ID: 8XE9). The heavy and light chains are shown in orange and red ribbons, respectively; the epitope footprint is rendered in blue surface. Key energetic hotspots on the RBD—including R403, N405, T415, Y421, Y453, L455, F456, A475, G476, N487, S490, Y501, H505—are highlighted in orange. (**D**) Close-up view of the interface showing how these hotspot residues contribute to the overall binding architecture. (**E**) Detailed interactions at the antibody side: Heavy chain residues F27, Y33, F58, L99, and R102 form key contacts with the RBD, visualized as magenta sticks.

Using the conformational ensembles of the RBD-antibody complexes, we embarked on structure-based mutational analysis of the S protein binding with antibodies. To provide a systematic comparison, we constructed mutational heatmaps for the RBD interface residues of the S complexes with class I antibodies. Mutational scanning of BD55-1205 binding revealed a large binding interface and identified multiple distributed hotspot residues that anchor the interaction, including: R403, N405, T415, Y421, Y453, L455, F456, A475, G476, N487, S490, Y501, H505 (Figure 4A,C,D). The large binding interface and multiple hotspot anchors for BD55-1205 distinguish it from other Class I antibodies. This unique characteristic is reflected in the mutational scanning heatmap showing that strikingly most of the RBD binding interface residues in the complex with BD55-1205 emerged as relevant binding hotspots (Figure 4A). Residues T415, Y421, Y453, L455, F456, A475, G476, N487, S490, Y501, H505 form a critical hydrophobic core within the RBM, stabilizing the interaction through van der Waals forces and hydrophobic packing. Y41, Y353, L455, and F456 form the central anchoring hotspot cluster and residues Y501, H505 function as anchors, providing additional stability to the complex through both hydrophobic and electrostatic interactions. Only several interfacial sites (D420, K458, S459, K460, N477 and P486) in the XBB.1.5 RBD displayed significant tolerance to mutations due to moderate interactions with BD55-1205 (Figure 4A).

Mutational scanning identifies residues critical for both RBD stability and high-affinity binding, and destabilizing mutations highlight “hotspots” that are functionally indispensable for the interactions and RBD stability. We noticed that the largest destabilization changes upon BD55-1205 binding are induced in positions Y421 and Y489 that are part of the hydrophobic core and critical for RBD stability and ACE2 binding (Figure 4A). Interestingly, our results also showed that mutations in a critical F456 position including F486P (ΔΔG = 2.36 kcal/mol) and F456L (ΔΔG = 1.54 kcal/mol) and F456V (ΔΔG = 1.86 kcal/mol) can affect RBD stability and binding though not as severely as substitutions at positions Y421 and Y489 (Figure 4A). Y421, Y473 and Y489 are critical hotspots for BD55-1205 stability and binding, underscoring their importance in maintaining structural integrity and binding affinity. F456 plays a secondary role in stabilizing the complex, with mutations causing moderate destabilization. However, its contribution to hydrophobic packing is crucial for maintaining overall binding affinity. It was suggested that superior neutralizing ability of BD55-1205 against all variants (including the ones with F456L/V) can be attributed to strong interactions with the RBD in this region. We examined the details of mutational scanning data by profiling both RBD and BD55-1205 residues (Figure 4A,C,D). Mutational scanning of the heavy chain BD55-1205 interfacial residues highlighted the two major hotspots Y33 and R102 (Figure 4B). Y33 is the key hotspot for interactions with F456 and R102 makes contacts with L455. We noticed that heavy chain residues Y33, P53, W94, L99, and I101 make contacts with L455 on the RBD, while important heavy chain centers R31, N32, Y33, P53, and L99 make contacts with F456 in the complex (Figure 4B,E). Interestingly, the largest destabilization mutations in the heavy chain are associated with mutations Y33D/E/K/S/N/Q [62].

**Figure 5:**
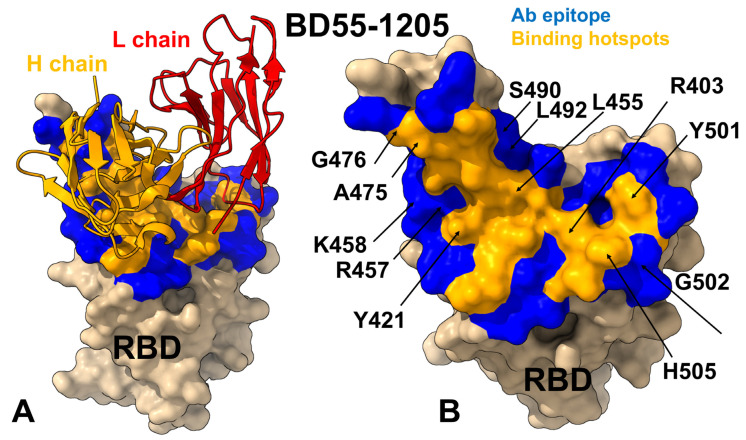
Structural mapping of BD55-1205 interactions with binding hotspots and backbone-contacting RBD residues. (**A**) Structure of BD55-1205 bound to the RBD (PDB ID: 8XE9). The heavy chain is shown in orange ribbons, light chain in red. The binding epitope on the RBD is displayed in blue surface, with binding hotspot residues in orange. (**B**) Close-up view of the interface showing how these backbone-interacting residues contribute to the overall binding architecture. The binding epitope residues involved in backbone-mediated contacts, including L455, R457, K458, A475, G476, S490, L492, and G502, are annotated.

Interestingly, the experimental structural studies by Cao and colleagues established that BD55-1205 leverages HCDR (T28, R31, N32, Y33, P53, R102) sites to form interactions with RBD backbone of residues L455, R457, K458, Q474, A475, G476, S490, L492, and G502 but not with F456 (Figure 5) [62]. Notably, the backbone-interacting residues R457, K458, G476, S490, L492 are not among dominant binding energy hotspots (Figure 5) and thus enable adaptability to evolving mutations in these positions without sacrificing the binding strength. The computational predictions are compared against the recent experimental data on average antibody escape scores (https://github.com/jbloomlab/SARS2-RBD-escape-calc/tree/main/Cao_data/JN1-evolving-antibody-response/data/DMS/antibody (accessed on May 29 2025)). These experimental data were generated with the escape calculator [103,104,105] and are reported in the update analysis (https://jbloomlab.github.io/SARS2-RBD-escape-calc/ (accessed on May 29 2025)) that included the latest yeast-display DMS data by Cao and colleagues [46].

**Figure 6:**
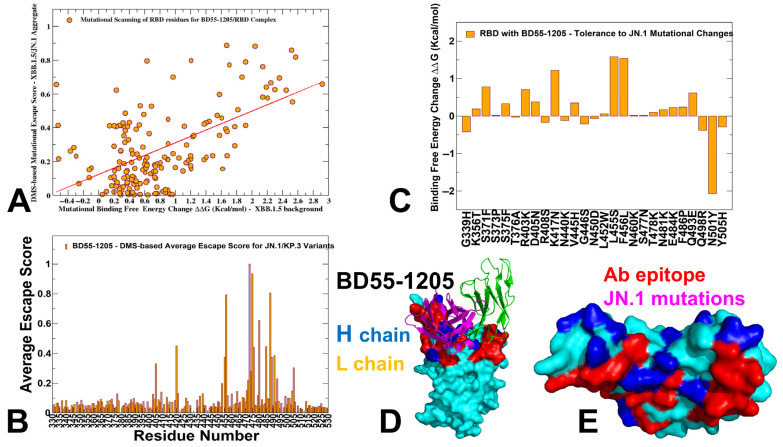
Comparative mutational sensitivity of BD55-1205 binding to the RBD. (**A**) Scatter plot comparing predicted binding free energy changes from computational mutational scanning with experimentally derived DMS escape scores for BD55-1205-bound RBD. Orange circles indicate strong agreement between computed destabilization effects and observed escape propensities (Pearson R = 0.57), validating the predictive power of the model. (**B**) Experimental escape scores show that mutations at residues Y473, A475, F456, and Y489 have the greatest impact on antibody binding, as reflected by elevated escape values. (**C**) Binding free energy changes induced by JN.1 variant-associated mutations. Destabilizing effects (positive ΔΔG values) are shown as orange bars, with the most disruptive mutations aligning to key functional residues in the RBD–antibody interface. (**D**) Structure of BD55-1205 bound to RBD. The heavy chain of BD55-1205 is in blue ribbons, and the light chain is in orange ribbons. The RBD is on the cyan surface and the binding epitope is shown in red. (**E**) RBD from the complex with BD55-1205. The binding epitope residues are in red, and the JN.1 mutational sites are highlighted in magenta.

By comparing the predicted binding free energy changes obtained from mutational scanning of BD55-1205 residues with the DMS-inferred mutational escape scores (Figure 6), we found an overall excellent agreement between the predicted and experimental data. The Pearson correlation coefficient R = 0.57 suggested statistically significant correspondence between the mutational scanning results and the DMS-derived escape scores. The largest binding free energy changes were observed for Y489T, F456G, Y489S, Y473G, F456N, F456P, Y473Q, Y473P and Y473K mutations, while the experimental mutational escape scores displayed the largest escape potential for mutations F456R, A475K, Y473G, F456G, Y473P (Figure 6A). Our predictions accurately predicted important escape mutations in Y489, Y473, A475 and F456 RBD sites. Interestingly, a near-complete loss of neutralization may indeed be associated with F456D, F456E, F456P, F456K, and F456R mutations in various backgrounds, but these mutations can also severely reduce ACE2 binding affinity and do not emerge in the evolving variants.

We also examined the experimentally determined average residue-based escape scores that emphasized major escape positions for BD55-1205 and included residues Y473, A475, C488, F456, C480, G485, G476, Y489, and L455 (Figure 6B). While C480 and C488 residues are not involved in the binding interface and are critical for preserving RBD integrity, other critical sites that are located at the binding interface, Y473, A475, F456, G476, Y489 and L455, were correctly singled out as mutational hotspots of BD55-1205 binding (Figure 4). In addition, some of the most detrimental mutations revealed by DMS data [46], including Y473A/S/K, A475K/R/H, F456G/N/P, are associated with the largest destabilization binding free energies in the computational mutational scanning (Figure 4). Since Y473 and Y489 are fundamentally important for RBD stability and ACE2 binding, the vulnerable sites, which may be exploited by the virus to escape BD55-1205, are A475 and F456.

We also analyzed how JN.1 variant mutations can influence the stability and affinity of the RBD/BD55-1205 complex (Figure 6C–E). Mutational profiling showed that L455S mutation induces the moderate destabilizing effect (ΔΔG ≈ 1.3 kcal/mol), highlighting its critical role as a hotspot for immune escape mutations across multiple variants. The high sensitivity of BD55-1205 to this residue underscores some reliance on hydrophobic interactions with L455. Another destabilizing mutation, F456L, leads to similar destabilization ΔΔG ≈ 1.1 kcal/mol (Figure 6C). F456 is also a key contact point between the RBD and BD55-1205, contributing to both hydrophobic packing and hydrogen bonding networks. Interestingly, the data suggested that JN.1 RBD mutations targeting hotspot residues L455, F456 could modulate immune escape by disrupting some key interactions. However, the broad epitope coverage and multi-node distributed hotspot architecture for BD55-1205 can rescue relatively moderate loss in van der Waals interactions and offer a degree of resilience that limits the effectiveness of single-site mutations.

In fact, our predictions agreed with DMS data suggesting that A475 and F456 mutations may cause localized loss in binding to BD55-1205. Indeed, A475V and L455F + F456L mutations emerged in several XBB.1.5 and BA.2.86 lineages, enabling escape from a broad range of class I antibodies. For instance, LF.7.2.1 demonstrates significantly increased immune evasion compared to XEC, primarily due to the A475V mutation, which enables the virus to escape neutralization by Class 1 antibodies [55]. Hence, the experimentally observed superior neutralizing capacity of BD55-1205 binding [62] can be attributed not to the lack of sensitivity to mutations in A475 and F456 but likely due to a broad and efficiently distributed network of hotspots and interactions that may enable it to sustain and partly mitigate the effect of mutations in these positions. The suggested distributed anchoring binding mechanism could contribute to BD55-1205 resilience to mutations. In this mechanism, even if one or two hotspot residues are mutated (L455, F456), the overall binding affinity would not be severely compromised due to the redundancy in stabilizing interactions.

Mutational scanning for BD-604 binding revealed a reduced breadth in the distribution of main hotspots, pointing to Y421, Y453, L455, and F456, Y489, R493, Y501, G502 and H505 as main energetic centers which are also critical for ACE2 engagement (Supporting Information, Figure S5A). Interestingly, the major destabilization mutations in BD-604 are generally similar to those in BD55-1205 and are associated with the protein stability effects, but the extent of destabilization is not as significant as for BD55-1205 (Supporting Information, Figure S5A,C,D). We also similarly evaluated mutational profiles for BD-604 and found that the most destabilizing mutations are associated with Y33 and Y102 and G26 (Supporting Information, Figure S5B). BD-604 exhibits greater tolerance for mutations of I28, S30, and S31 in HCDR1 that are more adaptable to substitutions. These residues interact with RBD residues Y473, Q474, A475, G476, Y501 and H505, suggesting a broader adaptability to certain mutations (Supporting Information, Figure S5E) but weaker cumulative interactions with the RBD residues. These differences may underscore the enhanced binding affinity and mutational resilience of BD55-1205 as compared to BD-604. Mutational scanning map suggested that RBD positions N417, N460 and F486 are moderately tolerant to substitutions while mutations in L455 and F456 are highly destabilizing. Due to less extensive interaction network formed by BD-604 could make it vulnerable to mutations in variants BA.4/BA5 and XBB.1.5. Indeed, the neutralizing ability of BD-604 showed considerable reduction in neutralization against BA.4/5, BQ.1.1, and XBB variants, highlighting the fact that mutations K417N, N460K in BA4/BA.5 and V445P, G446S, N460K, F486S, and F490S can reduce BD-604 binding, and these cumulative losses are far more detrimental for BD-604 activity than for BD55-1205.

For comparison, we also built mutational scanning heatmaps for other structures of antibody BD-604 bound with RBD (pdb id 7CHF, 7CH4), BD-604 bound with BA.2 RBD (pdb id 8HWT), BD-604 bound with BA.4 RBD (8HWS), RBD, BA.1 RBD and BA.2 RBD (Supporting Information, Figures S6 and S7). The results are very consistent among these complexes, emphasizing the critical triad of RBD hotspot positions Y453, L455, F456 as well as RBD residues Y473, Y489, Y501, G502 and H505.

We also constructed mutational heatmaps for the RBD interface residues of other S-RBD complexes with class I OMI-42 (Supporting Information, Figure S8). OMI-42 is susceptible to A475V and L455F + F456L mutations emergent in several XBB.1.5 and BA.2.86 lineages [106]. The mutational heatmap analysis showed that positions Y421, Y453, L455 and F456 emerged as key escape hotspots with Omi-42 (Supporting Information, Figure S8A,C,D). L455 and F456 positions are located at the epitope of RBD Class 1 antibodies and neutralization assays demonstrated that L455S mutation enables JN.1 to evade Class 1 antibodies. Mutational heatmaps data showed that effectively all modifications in L455, including L455S, can cause considerable loss in antibody binding. A secondary group of escape hotspots for OMI-42 included F486, N487, Y489, and Q493 positions (Supporting Information, Figure S8A,C,D). Mutational profiling of heavy chain residues of OMI-42 showed significant role of Y32, W53, F101, Y109 and Y110 (Supporting Information, Figure S8B,E). Compared to BD55-1205 and another ultrapotent antibody VIR-7229, OMI-42 forms fewer hydrogen bonds in the region near L455/F456, which may explain the markedly reduced neutralizing activity for XBB-descendant and JN.1-descendant variants harboring F456L [63]. Omi-42 binding is abrogated by several substitutions at F456 position in multiple backgrounds [63]. This contrasts with. BD55-1205, which has an exceedingly high barrier to viral resistance as the relative tolerance for epitope diversification is promoted by the extensive contacts with the RBD backbone.

P5S-2B10 and P5S-1H1 exhibit a nearly identical binding pose to the top face of the RBD, mimicking the binding mode of ACE2. These antibodies compete directly with ACE2 for binding. Mutational scanning of P5S-1H1 revealed key hotspots Y421, Y453, L455, F456 and H505, while positions K417, Y473, Y489 and N501 may be less sensitive to mutations (Supporting Information, Figure S9A,C,D). The analysis of P5S-1H1 contact residues pointed to Y33, Y52, F58, L99, and Y102 as important hotspot centers (Supporting Information, Figure S9B,E). The mutational heatmaps for P5S-2B10 are generally similar and revealed positions Y421, Y453, L455, F456 and H505 as major hotspots (Supporting Information, Figure S10A,C,D) while the corresponding energetic anchors on the antibody correspond to Y33, F58, Y100 but Y102 position is more tolerant and less energetically important (Supporting Information, Figure S10B–E).

To summarize, despite targeting very similar epitopes and sharing comparable binding modes, BD55-1205, BD-604, OMI-42, P5S-1H1, and P5S-2B10 exhibit subtle yet critical differences in their interaction patterns with the RBD. These distinctions arise from variations in epitope composition, reliance on specific residues, adaptability to mutations, and structural dynamics, collectively shaping their efficacy, breadth, and resilience to viral evolution. The comparison between BD55-1205 and other neutralizing class I antibodies further underscored the most striking difference—a large and broadly distributed footprint of strong hotspots for BD55-1205. BD55-1205 leverages a broad network of backbone-mediated hydrogen bonds with RBD residues and features a more extensive set of strong binding hotspots, thereby rescuing potential losses and enhancing adaptability to mutations in commonly shared positions L455 and F456. The mutational scanning analysis underscores the subtle differences in binding mechanisms employed by class I antibodies targeting remarkably similar epitopes but exerting distinct energetic patterns with the RBD. Importantly, the results suggested that major drivers of immune escape for this class I antibodies correspond to L455 and F456 sites that undergo mutations in the JN.1, KP.2 and KP.3 variants, enabling evolution through the enhanced immune escape.

### 3.4. MM-GBSA Computations of the Binding Energetics and Residue-Based Decomposition Analysis for Class I Antibody-RBD Complexes: Broadly Distributed Footprint of Multiple Binding Hotspots Determines Unique Neutralization Profile of BD55-1205

**Figure 7:**
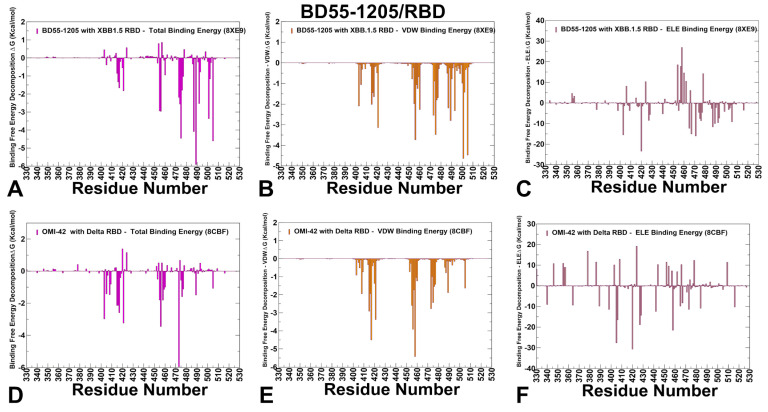
The residue-based decomposition of the binding MM-GBSA energies for BD55-1205 and OMI-42 binding. The residue-based decomposition for BD55-1205/RBD binding shows the total binding energy (**A**), van der Walls contribution to the total MM-GBSA binding energy (**B**) and the electrostatic contribution to the total binding free energy (**C**). MM-GBSA contributions are evaluated using 1000 samples from the simulations of BD55-1205 complex with RBD, pdb id 8XE9. The residue-based decomposition for OMI-42/RBD binding shows the total binding energy (**D**), van der Walls contribution to the total MM-GBSA binding energy (**E**) and the electrostatic contribution to the total binding free energy (**F**). MM-GBSA contributions are evaluated using 1000 samples from simulations of OMI-42 complex, pdb id 8CBF. Residue-based binding free energy values are shown for total energies as magenta-colored filled bars, van der Waals contributions as orange-colored bars and electrostatic contributions as light-brown colored bars.

We utilized conformational equilibrium ensembles derived from atomistic reconstruction of CABS trajectories to compute the binding free energies for the RBD-antibody complexes using the MM-GBSA method. This approach allowed us to identify key binding hotspots and quantify the roles and synergies of van der Waals and electrostatic interactions in the binding mechanism. The key energetic centers of BD55-1205 binding with the RBD are broadly spread and correspond to residues Y489, H505, A475, Y421, L492, Y501, F456, L455, G476 and N487 displaying the largest total binding energies (Figure 7A) The analysis of van der Waals interactions showed that the largest contributions correspond to residues H505, L455, F456, A475, Y421, Y489, N487, and Y473 (Figure 7B). For example, F456 and L455 contribute significantly to hydrophobic interactions, as evidenced by their favorable van der Waals energies (−3.88 kcal/mol and −2.04 kcal/mol, respectively) (Figure 7B). These results are consistent with the mutational scanning analysis of BD55-1205 interactions with the RBD, which revealed positions Y421, Y453, L455, F456, A475, G476, N487, S490, Y501, H505 as dominant energetic centers. MM-GBSA results emphasized the role of H505, Y501, F456, A475, and Y489 sites due to their favorable van der Waals forces and hydrophobic packing (Figure 7B). The strongest electrostatic interactions are formed with R403, E471, D467 and N487 residues, where R403 and N487 positions contribute favorably through synergy of the van der Waals and electrostatic contributions (Figure 7C). Overall, the hydrophobic contacts provide dominant contribution to the total binding energy and are particularly favorable and synergistic with other contributions for Y421, F456, Y473, A475, Y489 and H505 positions (Figure 7A–C). Strikingly, our predictions are in excellent agreement with DMS escape profiles (based on XBB.1.5 RBD) for BD55-1205, showing that major immune escape sites are Y473, A475, F456, Y489 [62].

It is important to note that BD55-1205 leverages interactions with the RBD backbone in positions L455, R457, K458, Q474, A475, G476, S490, L492, and G502 [62]. Only some of these residues, A475, G476 and L492, are among strong energetic centers and the spectrum of binding energy hotspots appeared to be much broader and included also Y421, Y453, F456, Y501, and H505 residues. This analysis suggested that the “backbone-interacting” RBD positions could provide a second “line of defense” against BD55-1205 resistance as mutations in convergent evolution sites L455 and F456 may be potentially rescued and compensated by sustainable backbone-based interactions (Figure 7A–C). Based on the MM-GBSA analysis and mutational scanning data, we further reinforced our main finding that the unique binding mechanism of BD55-1205 is determined by its broadly distributed network of binding hotspots that may define a high barrier to viral resistance. While some hotspots are important for ACE2 binding and therefore functionally and evolutionary constrained, tolerance for mutations in the L455/F456 region can be enabled through extensive interactions of broadly distributed RBD hotspots.

The MM-GBSA calculations for another class I antibody OMI-42 identify several residues as critical hotspots for OMI-42 binding: Y473, F456, Y421, R403, K417, T415 and G416, where Y473 and F456 dominate the total binding energy (Figure 7D). The breakdown of the binding free energy components revealed that F456, K417, Y421, T415, Y473, L455 and A475 contribute strongly to van der Waals stabilization (Figure 7E), while the electrostatic interactions favor D420, D405, K458, D427, but their total contributions are relatively moderate due to solvation effects (Figure 7F). Overall, the MM-GBSA analysis revealed strong dominant contributions of F456, Y421, T415, Y473, L455 and A475 to binding and may be susceptible to mutations in these positions. Although the binding epitope for OMI-42 is generally similar to BD55-1205, the footprint of major binding hotspots is somewhat different and more localized compared to BD55-1205, making it more susceptible to mutations at critical positions L455, F456, and A475 (Figure 7D–F). The results confirmed the experimental finding that KP.2 and KP.3 harboring the F456L mutation can significantly impair the neutralizing activity of Omi-42 [45]. Indeed, the functional experiments showed that Omi-42 is susceptible to A475V and L455F + F456L mutations that emerged in several XBB.1.5 and BA.2.86 lineages and may be partly affected by D405N and R408 [100].The recently circulating evasive variants, including HK.3.1, JD.1.1, and JN.1, could efficiently escape OMI-42 due to their escape mutations (L455F + F456L) of HK.3.1 and the additional A475 V mutation carried by JD.1.1 [107,108]. In summary, the L455 and F456 positions appear to be particularly critical vulnerabilities for OMI-42, with mutations at these sites providing significant escape potential for the virus.

**Figure 8:**
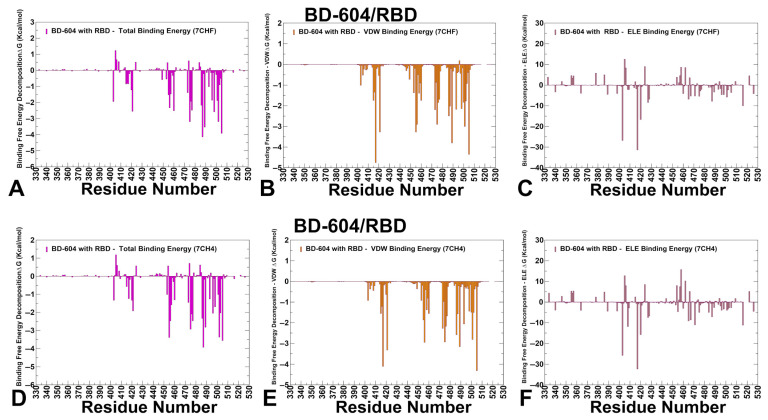
The residue-based decomposition of the binding MM-GBSA energies for BD-604 binding. The residue-based decomposition for BD-604/RBD binding shows the total binding energy (**A**), van der Walls contribution to the total MM-GBSA binding energy (**B**) and the electrostatic contribution to the total binding free energy (**C**). MM-GBSA contributions are evaluated using 1000 samples from the simulations of BD-604 complex with RBD, pdb id 7CHF. The residue-based decomposition for BD-604/RBD binding shows the total binding energy (**D**), van der Walls contribution to the total MM-GBSA binding energy (**E**) and the electrostatic contribution to the total binding free energy (**F**). MM-GBSA contributions are evaluated using 1000 samples from simulations of OMI-42 complex, pdb id 7CH4. Residue-based binding free energy values are shown for total energies as magenta-colored filled bars, van der Waals contributions as orange-colored bars and electrostatic contributions as light-brown colored bars.

MM-GBSA for BD-604 binding emphasized the role of N487, Y505, Y489, A475, G502, Q498, K417, Y421 and N460 residues to the total binding energy (Figure 8A). The van der Waals interactions were strongest for K417, Y505, Y489, Y421 and L455 (Figure 8B). The electrostatic contacts were favorable K417, R403 and D420 but were often largely offset by unfavorable solvation (Figure 8C). For comparison, we also examined MM-GBSA energies using an ensemble obtained from simulations of another BD-604 complex with RBD (Figure 8D–F). As may be expected, the results revealed similar patterns, highlighting N487, L455, F456, A475, H505 and Y489 as key binding hotspots (Figure 8D–F). BD-604 exhibits a more localized binding mechanism, focusing its energy on energetically dominant residues such as Y421, Y453, L455, F456, Y489, G502, and H505 that include functionally and evolutionary constrained positions indispensable for RBD stability and ACE2 binding (Y421, Y453, Y489, G502, H505) along with convergent evolution centers L455 and F456. BD-604 also relies more heavily on side-chain contacts rather than backbone-mediated hydrogen bonds, making it less adaptable to structural changes in the RBD. The experimentally measured escape profiles and neutralizing ability of BD-604 showed considerable reduction in neutralization against BA.4/5, BQ.1.1, and XBB variants, highlighting the fact that mutations K417N, N460K in BA4/BA.5 and V445P, G446S, N460K, F486S, and F490S can reduce BD-604 binding [100].

**Figure 9:**
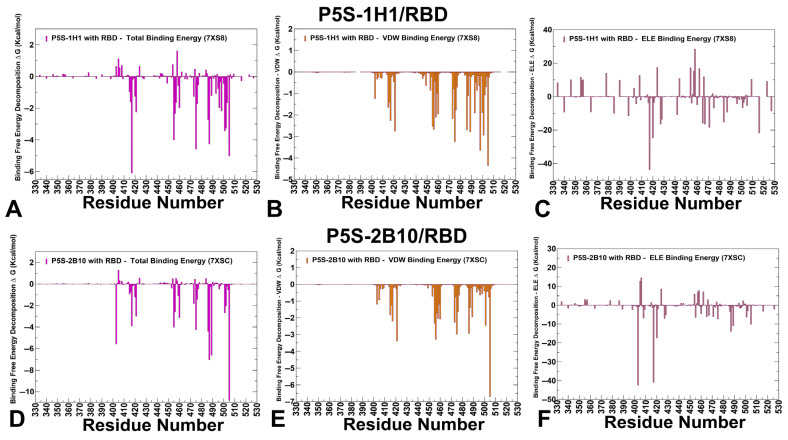
The residue-based decomposition of the binding MM-GBSA energies for P5S-1H1 and P5S-2B10 binding. The residue-based decomposition for P5S-1H1/RBD binding shows the total binding energy (**A**), van der Walls contribution to the total MM-GBSA binding energy (**B**) and the electrostatic contribution to the total binding free energy (**C**). MM-GBSA contributions are evaluated using 1000 samples from the simulations of P5S-1H1 complex with RBD, pdb id 7XS8. The residue-based decomposition for P5S-2B10/RBD binding shows the total binding energy (**D**), van der Walls contribution to the total MM-GBSA binding energy (**E**) and the electrostatic contribution to the total binding free energy (**F**). MM-GBSA contributions are evaluated using 1000 samples from simulations of P5S-2B10 complex, pdb id 7XSC. Residue-based binding free energy values are shown for total energies as magenta-colored filled bars, van der Waals contributions as orange-colored bars and electrostatic contributions as light-brown colored bars.

P5S-1H1 and P5S-2B10 antibodies belong to Class I neutralizing antibodies, which target epitopes overlapping with the ACE2-binding site. MM-GBSA analysis of P5S-1H1 revealed a number of key hotspots including K417, Y505, A475, N487, L455, F486, N501, G502 (Figure 9A). The strongest residues displaying favorable van der Waals contacts are Y505, A475, Y489, Y421, F456 and F486 (Figure 9B). K417 exhibits strong van der Waals and electrostatic interactions while A475, L455 and F486 contribute primarily through van der Waals interactions, stabilizing the hydrophobic core within the receptor-binding motif (RBM) (Figure 9B). Interestingly, unlike BD1205 and BD-604, this antibody binding is less dependent on F456 site but strongly relies on electrostatic contacts with K417 (Figure 9C) and hydrophobic interactions with A475, Y489, Y505 and F486. As a result, mutations in K417, Y505 and F486 seen in various Omicron variants can reduce the neutralizing activity of P5S-1H1 [62]. These results suggested that P5S-1H1 can rely significantly on K417 and could be affected by mutations in K417. Additionally, P5S-1H1 makes important contacts with L455, F486 residues that are mutated in various Omicron variants, including XBB.1.5, SLip, FLiRT, and KP.2 variants.

Combined with a narrower spectrum of binding hotspots compared to BD55-1205, these data suggest that P52S-1H1 can be escaped by variants invoking mutations in positions K417, L455 and F486 (Figure 9A–C). For P5S-2B10 the major binding hotspots are Y505, N487, Y489, R403, F486, A475, L455 and K417 (Figure 9D–F). The favorable van der Waals contacts are dominated by Y505, Y421, F456, A475 and F486 residues (Figure 9E), while electrostatics is driven by R403 and K417 (Figure 9F) While there are some notable differences in relative order of major energetic centers, the overall interaction pattern is similar reflecting strong dependence on conserved sites indispensable for stability and ACE2 binding as well as importance of L455, F456, A475, F486 and K417 (Figure 9D–F).

The general insights obtained from MM-GBSA analysis and mutational scanning results highlight the role of convergent evolution, trade-offs between breadth and specificity in immune escape and functional stability constraints. L455 and F456 consistently emerge as dominant escape hotspots across all antibodies, enabling viral evolution through enhanced immune evasion. Antibodies with broad epitope coverage, such as BD55-1205, exhibit greater resilience to mutations owing to a larger and more broadly distributed spectrum of major binding hotspots. We suggest that through this distributed hotspot mechanism, BD55-1205 can sustain strong binding affinity and mitigate the effects of mutations in the latest variants emerging in positions L455, F456 and A475.

## 4. Discussion

Through an integrative approach combining structural analysis, mutational profiling, coarse-grained simulations, and MM-GBSA computations, we uncover critical insights into the binding epitopes, energetic contributions, and dynamic signatures of class I antibodies such as BD55-1205, BD-604, OMI-42, P5S-1H1, and P5S-2B10. These results not only elucidate the diversity of binding mechanisms but also underscore the nuanced interplay between antibody specificity, mutational adaptability, and viral evolution. The dynamic signatures of the RBD induced by different class I antibodies reveal important distinctions in their functional profiles. BD55-1205 induces significant stabilization of key RBD regions, effectively locking the RBD into a rigid conformation that enhances stability and strengthens binding interactions. This rigidity likely contributes to BD55-1205’s ability to block ACE2 interactions and neutralize the virus. In contrast, BD-604 allows greater fluctuations in residues 450–475, suggesting a less constrained RBD conformation that may reduce its efficacy in blocking ACE2 binding. Intermediate behavior is observed for antibodies like P5S-1H1 and P5S-2B10, which stabilize the RBD to some extent but exhibit moderate fluctuations in regions like 490–505, indicating partial stabilization.

A central theme emerging from this work is the critical role of epitope breadth and interaction diversity in determining antibody resilience to mutations. BD55-1205 antibody exemplifies the advantages of broad epitope coverage and distributed hotspot mechanisms. By engaging an extensive network of residues across the RBD, BD55-1205 minimizes its dependence on individual side-chain conformations, allowing it to maintain robust binding even when key residues are mutated. This adaptability is particularly evident in its tolerance to mutations at positions such as L455 and F456, which severely compromise other antibodies. The ability of BD55-1205 to sustain cumulative interactions underscores the importance of targeting diverse epitopes through multiple interaction mechanisms, a strategy that enhances resistance to immune evasion while maintaining functional integrity.

The computational predictions generated through mutational scanning and MM-GBSA analysis demonstrate excellent agreement with experimental data on average antibody escape scores. The comparison among these antibodies underscores a broader design principle: targeting structurally conserved yet dynamically influential networks rather than isolated residues increases the likelihood of durable neutralization. BD55-1205 achieves this through synergistic effect of hydrophobic packing and electrostatic interactions, ensuring that no single mutation fully abrogates binding. In contrast, antibodies BD-604 and OMI-42 derive much of their binding stability from fewer, more concentrated contacts, rendering them less adaptable to change. While BD-604 forms strong interactions with specific residues, its narrower focus renders it more susceptible to escape mutations at critical positions. For instance, mutations such as K417N, N460K, V445P, and F486S significantly reduce BD-604 efficacy against variants like BA.4/BA.5, BQ.1.1, and XBB. These findings highlight the trade-offs between specificity and adaptability in antibody design, emphasizing the need for strategies that balance high-affinity binding with resilience to antigenic variation. The weaker cumulative interactions observed for BD-604 also underscore the limitations of relying heavily on side-chain contacts, which are more sensitive to structural changes in the RBD. The shared vulnerabilities of P5S-1H1 and P5S-2B10 to mutations at L455 and F456 highlight their susceptibility to convergent evolutionary pressures. While they may retain efficacy against certain variants, their narrower spectrum of binding hotspots makes them less robust in the face of antigenic drift.

Ultimately, this study highlights the evolutionary constraints shaping both viral adaptation and immune recognition. Mutations at key interface residues often come with a fitness cost, limiting the virus’s ability to evade all antibodies simultaneously. However, certain positions—notably L455 and F456—have become epistatic hubs, where mutations can both reduce antibody binding and maintain ACE2 compatibility, enabling immune escape without fitness loss. From a therapeutic perspective, these findings advocate for rational antibody cocktails that combine broad-spectrum binders like BD55-1205 with potent, narrowly focused antibodies, using intermediate binders as bridging agents. Such combinations would maximize coverage and durability, while minimizing the risk of complete escape.

## 5. Conclusions

This study provides a comprehensive mechanistic framework for understanding how Class I neutralizing antibodies BD55-1205, BD-604, OMI-42, P5S-1H1, and P5S-2B10 engage the RBD of the S protein and how their binding modes influence susceptibility to immune escape. Through multiscale modeling, we reveal that despite overlapping epitopes, these antibodies exhibit distinct interaction patterns that shape their neutralization breadth, binding stability, and evolutionary resilience. BD55-1205 emerges as a model of structural robustness, leveraging a broadly distributed network of contacts that includes both side-chain and backbone-mediated interactions. This extensive engagement allows it to maintain high-affinity binding even in the presence of mutations at key interface residues such as L455 and F456, where other antibodies lose efficacy. In contrast, antibodies BD-604 and OMI-42 demonstrate more localized binding footprints and a greater reliance on side-chain interactions, which are inherently more sensitive to substitutions. Mutations such as K417N, N460K, V445P, and F486S, now common in Omicron subvariants, significantly impair their binding, underscoring the vulnerability of narrower interaction networks under immune pressure. P5S-1H1 and P5S-2B10 fall into an intermediate category, offering moderate stabilization of the RBD but lacking the full functional redundancy seen in BD55-1205. These antibodies retain some degree of potency across variants but face growing challenges from convergent mutations at L455 and F456, reinforcing the idea that escape pathways often converge on functionally constrained sites, where substitutions balance ACE2 compatibility with reduced antibody recognition. The observed critical role of convergent mutational hotspots L455 and F456 reflects the intense selective pressures driving viral adaptation. These positions serve as dominant escape hotspots, underscoring their dual role in mediating both host–virus interactions and immune recognition. This duality highlights the evolutionary trade-offs the virus must navigate—preserving entry efficiency while evading immune detection. Our results also emphasize the importance of targeting structurally invariant yet dynamically influential regions of the RBD, rather than focusing on mutation-prone surface residues. By integrating structural mapping, mutational scanning, and dynamic profiling, we demonstrate that antibody durability is not solely determined by static epitope conservation, but also by how binding reshapes the conformational landscape of the RBD and distributes energetic contributions across the interface. By bridging computational predictions with experimental observations, this work supports the development of rational strategies to anticipate and counteract immune evasion, including engineering antibodies that mimic distributed hotspot mechanisms.

## Data Availability

The original contributions presented in this study are included in the article/supplementary material. Crystal structures were obtained and downloaded from the Protein Data Bank (https://www.rcsb.org/ (accessed on 1 May 2025)). The rendering of protein structures was performed with interactive visualization program UCSF ChimeraX package (https://www.rbvi.ucsf.edu/chimerax/ (accessed on 1 May 2025)) and Pymol (https://pymol.org/2/ (accessed on 1 May 2025)). All mutational heatmaps were produced using the developed software that is freely available at https://alshahrani.shinyapps.io/HeatMapViewerApp/ (accessed on 5 May 2025).

## Supplementary Materials

The following supporting information can be downloaded at: https://www.mdpi.com/article/10.3390/v17081029/s1. Figure S1 presents COVID-19 Variant Dashboard—Global coverage. Figure S2 presents an overview of the phylogenetic analysis and divergence of Omicron variants. Figure S3 describes the evolutionary tree of current SARS-CoV-2 clades. Figure S4 presents representative conformations of ensembles of the RBD complexes and binding epitopes for class I antibodies obtained from the equilibrium trajectories. Figure S5 presents mutational sensitivity analysis and structural mapping of BD-604 binding to RBD. Figure S6 describes structural organization of the RBD complexes and binding epitopes for structures of BD-604 antibody with RBD and various Omicron variants. Figure S7 presents the results of dynamic mutational profiling of the RBD intermolecular interfaces in the different structures of RBD complexes with BD-604. Figure S8 presents detailed results of the dynamic mutational profiling of the RBD intermolecular interfaces in the RBD complex with OMI-42. Figure S9 presents detailed results of the dynamic mutational profiling of the RBD intermolecular interfaces in the RBD complex with P5S-1H1 antibody. Figure S10 presents detailed results of the dynamic mutational profiling of the RBD intermolecular interfaces in the RBD complex with P5S2B10 antibody. Table S1 lists Omicron variants with assigned clade annotation. Table S2 describes the mutational landscape of various Omicron variants. Table S3 lists the intermolecular interfacial residues and contacts in the structure of BD55-1205 complex with RBD. Table S4 lists the intermolecular interfacial residues and contacts in the structure of BD-604 complex with RBD. Table S5 lists the intermolecular interfacial residues and contacts in the structure of OMI-42 complex with RBD. Table S6 lists the intermolecular interfacial residues and contacts in the structure of P5S-1H1 complex with RBD. Table S7 lists the intermolecular interfacial residues and contacts in the structure of P5S-2B10 complex with RBD.
