# Supplementary material for: Multiscale Modeling and Dynamic Mutational Profiling of Binding Energetics and Immune Escape for Class I Antibodies with SARS-CoV-2 Spike Protein: Dissecting Mechanisms of High Resistance to Viral Escape Against Emerging Variants
Source: Viruses. 2025 Jul 23;17(8):1029. doi: 10.3390/v17081029 (PMC12390076; doi:10.3390/v17081029)
Supplement: Supplementary file 1 [file viruses-17-01029-s001.zip › viruses-3717688-supplementary/SUPPLEMENTARY MATERIALS/SUPPLEMENTARY_MATERIALS_VIRUSES_REVISION.pdf]

Supplementary Materials

Multiscale Conformational Landscaping and Dynamic Mutational Profiling of Binding Energetics and Immune Escape for Potent Class I Antibodies with SARS-CoV-2 Spike Protein: Dissecting Molecular Determinants of Uniquely Broad Neutralization Against Existing Variants for BD55-1205 Antibody

Mohammed Alshahrani [1], Vedant Parikh [1], Brandon Foley [1], and Gennady Verkhivker [1,2*]

[1] Keck Center for Science and Engineering, Graduate Program in Computational and Data Sciences, Schmid College of Science and Technology, Chapman University, Orange, CA 92866, United States of America alshahrani@chapman.edu (M.A); vedpar31@gmail.com (V.P.); brfoley@chapaman.edu (B.F.); verkhivk@chapman.edu (G.V).

[2] Department of Biomedical and Pharmaceutical Sciences, Chapman University School of Pharmacy, Irvine, CA 92618, United States of America

* Correspondence: verkhivk@chapman.edu; Tel.: +1-714-516-4586 (G.V)

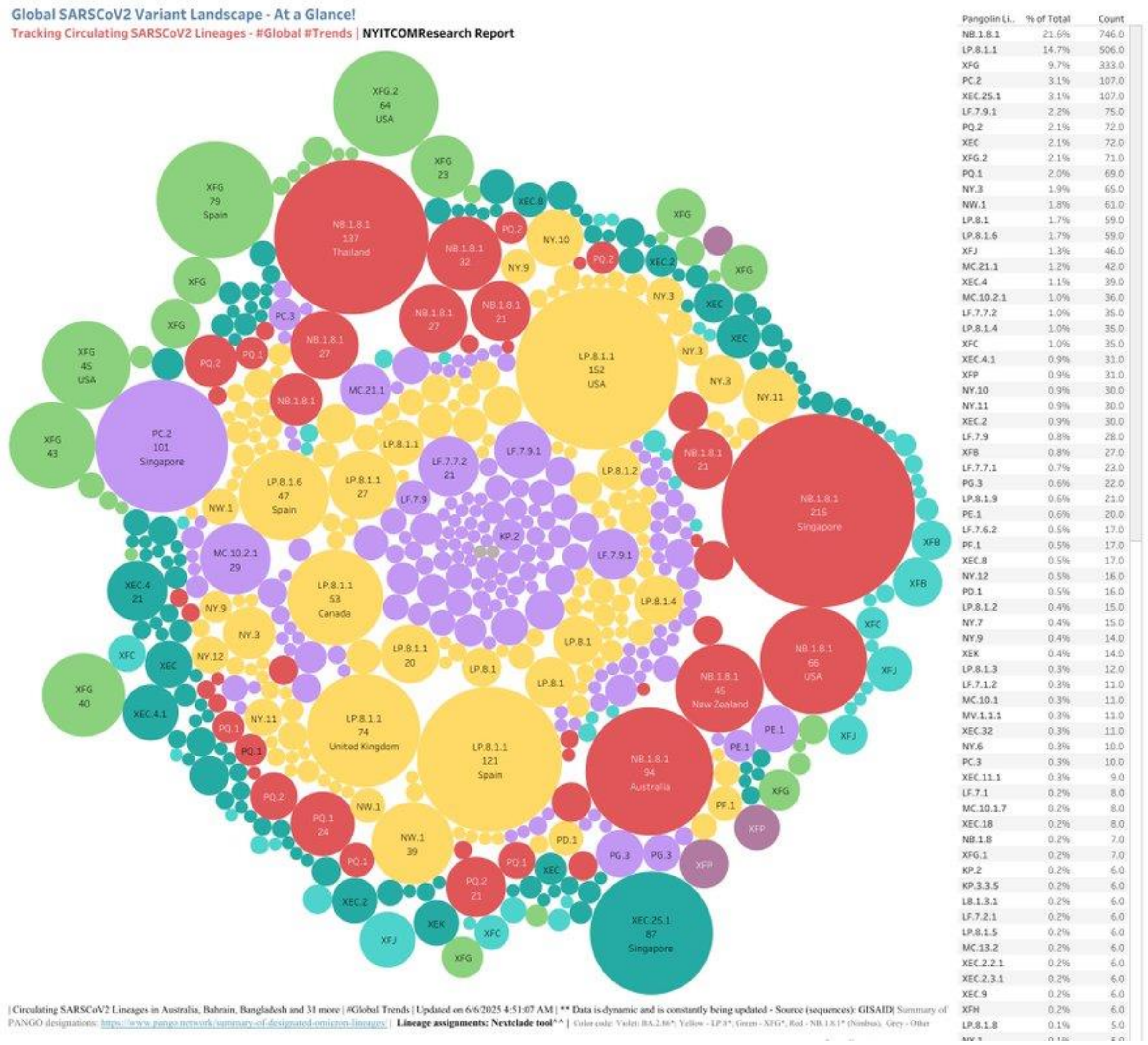

Figure S1. COVID-19 Variant Dashboard – Global coverage . Top lineages are NB.1.8.1 (21.6%), LP.8.1.1 (14.7%), XFG (9.7%), PC.2 (3.1%), XEC.25.1 (3.1%), LF.7.9.1 (2.2%), PQ.2 (2.1%), XEC (2.1%) XFG.2 (2.1%) (https://public.tableau.com/app/profile/raj.rajnarayanan/viz/USAVariantDB/VariantDashboard)

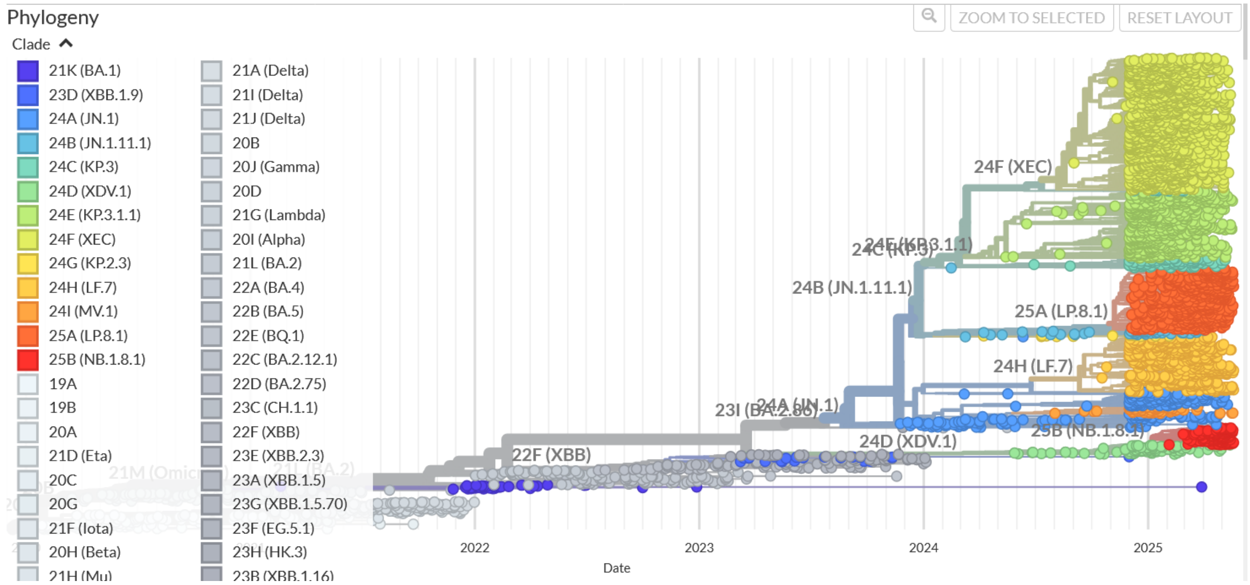

Figure S2. An overview of the phylogenetic analysis and divergence of Omicron variants. The graph is generated using Nextstrain, an open-source project for real time tracking of evolving pathogen populations (https://nextstrain.org/)

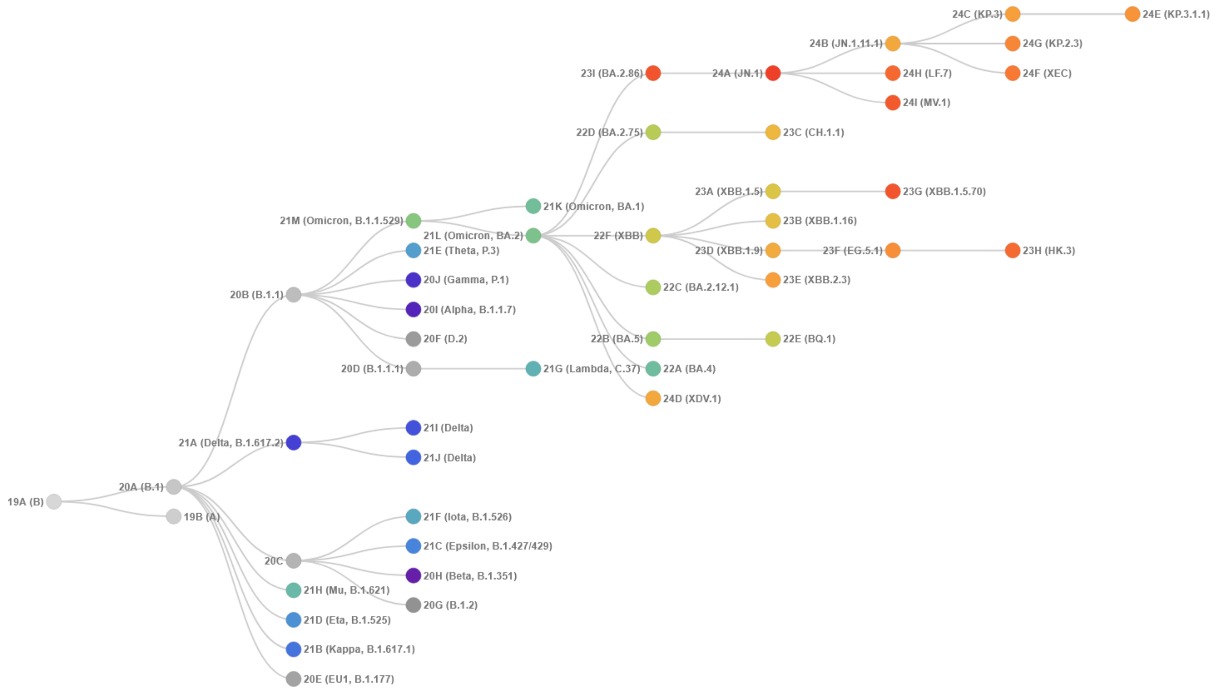

Figure S3. The evolutionary tree of current SARS-CoV-2 clades. The graph is generated using Nextstrain, an open-source project for real time tracking of evolving pathogen populations (https://nextstrain.org/). The clade 22F corresponds to XBB, 23A corresponds to XBB.1.5, 23G corresponds to XBB.1.5.70 (B.1.5+L455F+F456L) variant, 24C is KP.3 and 24E is KP.3.1.

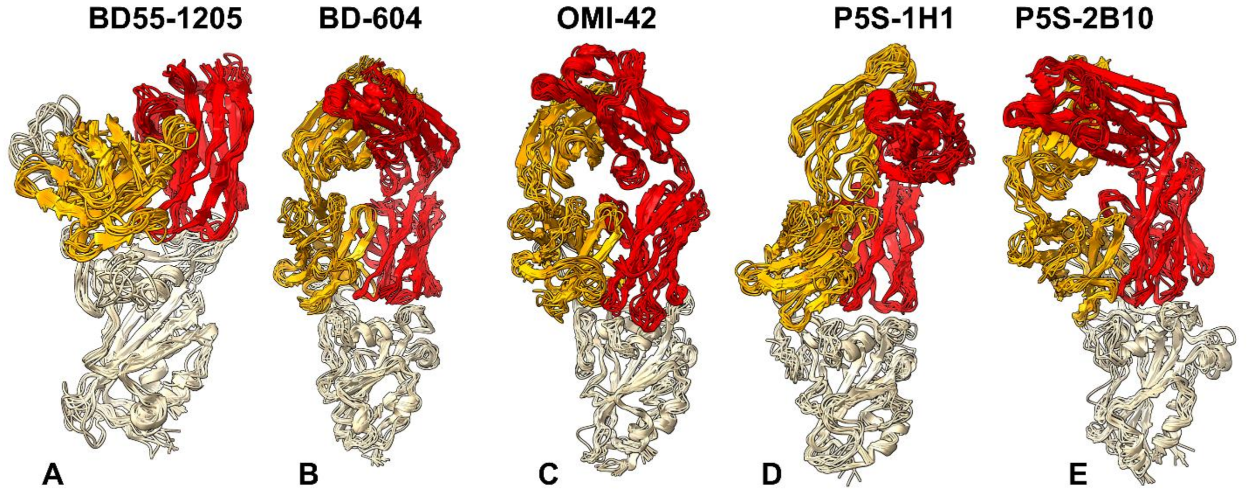

Figure S4. Conformational ensembles of the RBD complexes and binding epitopes for class I antibodies using representative conformations from the equilibrium trajectories. (A) The structure of BD55-1205 with XBB.1.5 RBD (pdb id 8XE9). The heavy chain in orange ribbons, the light chain in red ribbons. (B) The structure of BD-604 bound with BA.2 RBD (pdb id 8HWT). The heavy chain in orange ribbons, the light chain in red ribbons. (C) The structure of OMI-42 bound with Delta RBD (pdb id 8CBF). The heavy chain in orange ribbons, the light chain in red ribbons. (D) The structure of P5S-1H1 bound with RBD (pdb id 7XS8). The heavy chain in orange ribbons, the light chain in red ribbons. (IE) The structure of P5S-2B10 bound with RBD (pdb id 7XSC). The heavy chain in orange ribbons, the light chain in red ribbons.

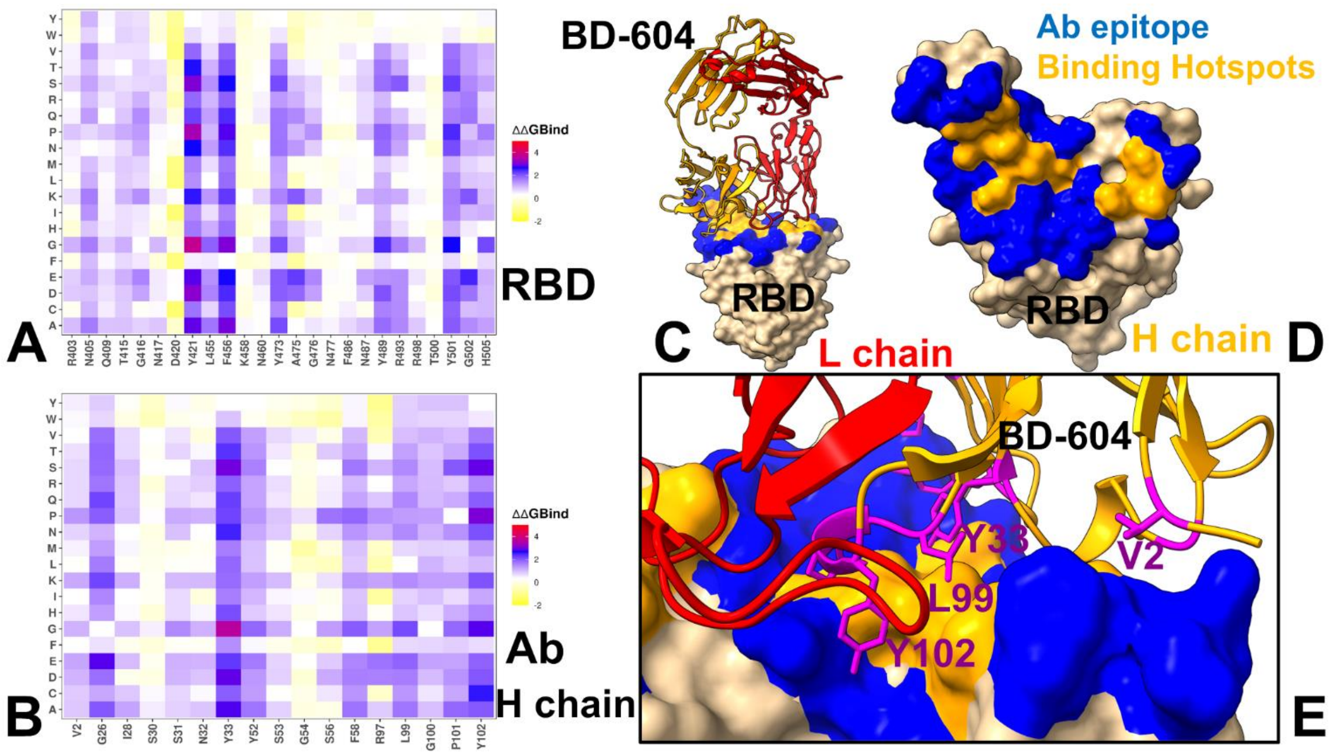

Figure S5. Mutational sensitivity and structural mapping of BD-604 binding to BA.2 RBD. (A) Ensemble-based mutational scanning of interfacial RBD residues in the BD-604 complex (PDB ID: 8HWT) reveals key destabilizing mutations, particularly at positions Y421, L455, F456, Y489, and H505 , which are central to antibody engagement. (B) Complementary heatmap of heavy chain residue contributions identifies V2, Y33, L99, and Y102 as dominant hotspots for RBD binding, with mutation-induced disruption correlating with loss of neutralization potency. (C) Structural view of BD-604 bound to BA.2 RBD shows the epitope footprint in blue surface. Critical interaction sites — including Y421, Y453, L455, F456, Y489, G502, and H505 — are highlighted in orange to reflect energetic importance. (D) Close-up surface representation of the interface emphasizes the spatial clustering of these hotspot residues, underscoring their collective role in stabilizing the antibody–RBD interaction. (E) Detailed visualization of the binding interface, with heavy chain residues V2, Y33, L99, and Y102 shown as magenta sticks. These residues form key contacts with the RBD, and substitutions at these positions significantly reduce binding affinity.

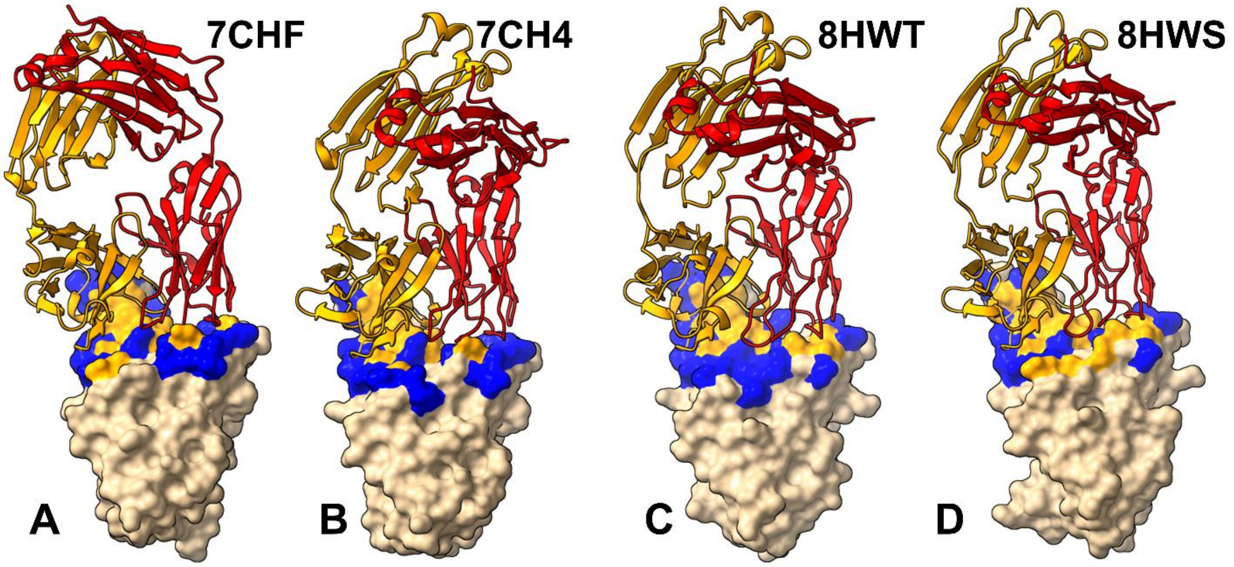

Figure S6. Structural organization of the RBD complexes and binding epitopes for structures of BD-604 antibody with RBD and various Omicron variants. The structures of BD-604 complex with RBD, pdb id 7CHF, (A) and pdb id 7CH4 (B), BD-604 complex with BA2 RBD, pdb id 8HWT (C) and BD-604 complex with BA.4/BA.5 RBD, pdb id 8HWS (D). The heavy chain in orange ribbons, the light chain in red ribbons. (D) The RBD and binding epitope footprint for BD-604. The binding epitope residues are shown in blue surface.

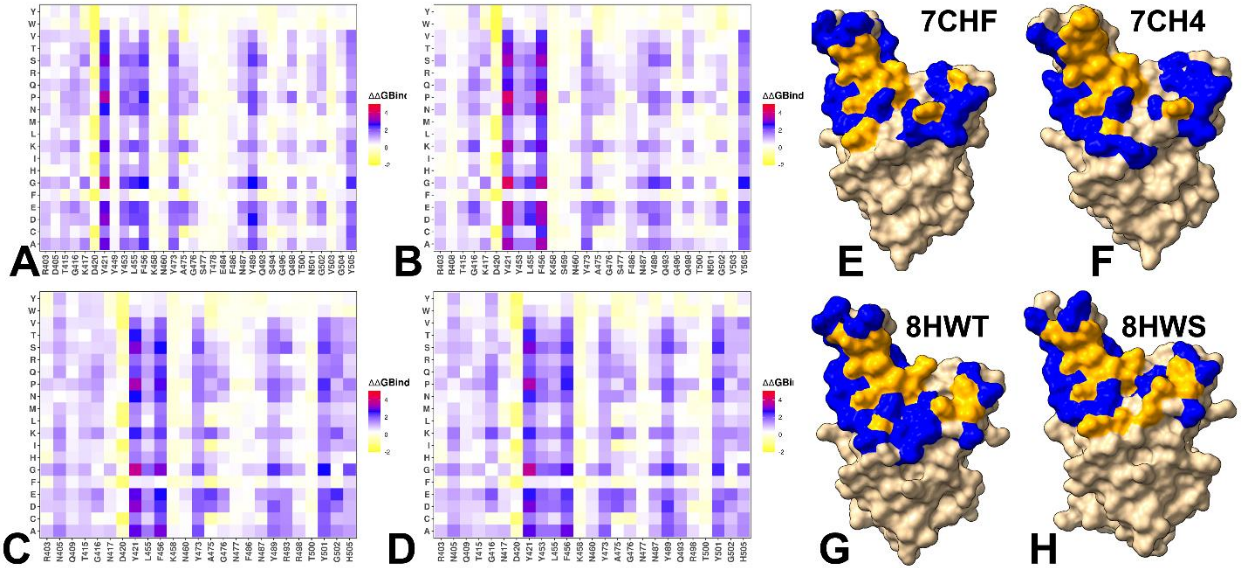

Figure S7. Ensemble-based dynamic mutational profiling of the RBD intermolecular interfaces in the different structures RBD complexes with BD-604. (A,B). The mutational scanning heatmaps are shown for the interfacial RBD residues in the BD-604 complex with RBD, pdb id 7CHF (A), BD-604 complex with RBD, pdb id 7CH4 (B), BD-604 complex with BA.2 RBD, pdb id 8HWT (C), BD-604 complex with BA.4/BA.5 RBD, pdb id 8HWS (D). The structures of BD-604 to RBD, pdb id 7CHF (E), pdb id 7CH4 (F), pdb id 8HWT (G), pdb id 8HWS (H). The heavy chain of BD-604 is in orange ribbons, and light chain is in red ribbons. The binding epitope is shown in blue surface and the positions of the RBD binding energy hotspots are shown in orange-colored surface.

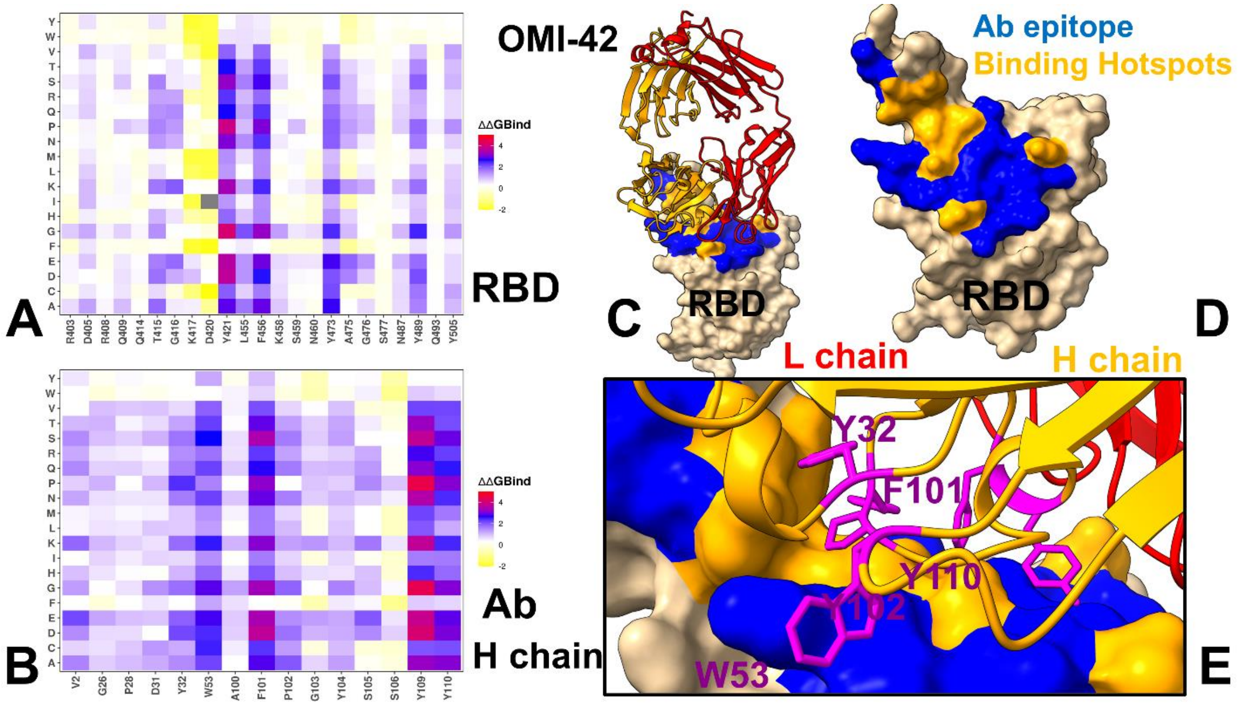

Figure S8. Ensemble-based dynamic mutational profiling of the RBD intermolecular interfaces in the RBD complex with OMI-42. (A,B). The mutational scanning heatmaps are shown for the interfacial RBD residues (A) and interfacial heavy chain residues of OMI-42 (B). (C) The structure of OMI-42 bound to RBD (pdb id 8CBF). The heavy chain of OMI-42 is in orange ribbons, and light chain is in red ribbons. The binding epitope is shown in blue surface and the positions of the RBD binding energy hotspots are shown in orange-colored surface. (D). RBD from the complex with OMI-42. The binding epitope residues are in blue surface and the binding interfacial RBD hotspots are in orange surface. (E) A closeup of the binding interface contacts of the OMI-42 hotspots from the heavy chain Y32, W53, F102 and Y110 . The heavy chain is in orange ribbons, light chain in red ribbons. The OMI-422 hotspots are shown in magenta sticks and annotated.

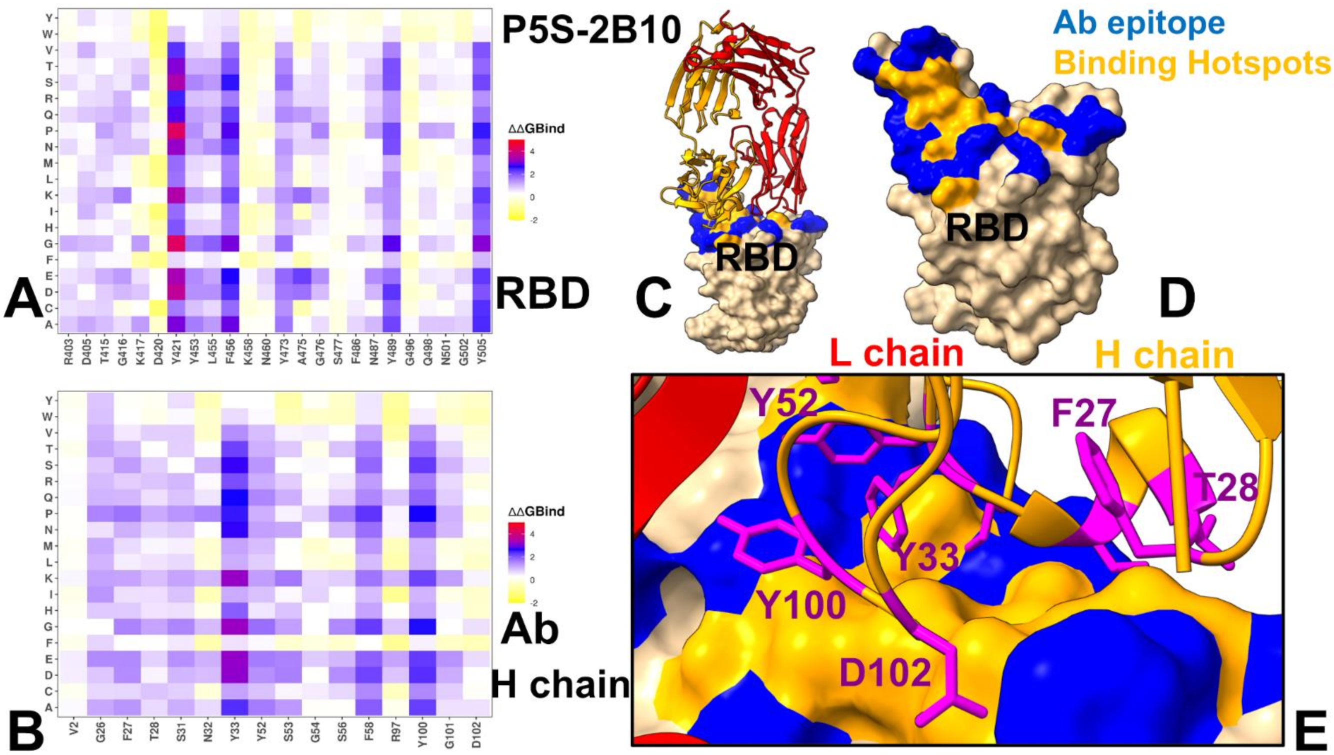

Figure S9. Mutational sensitivity and binding interface analysis of the P5S-1H1–RBD complex. (A, B) Dynamic mutational profiling was used to compute binding free energy changes upon residue substitutions at interfacial positions in the RBD and antibody heavy chain. The data highlight key residues that, when mutated, significantly destabilize the complex.(C) Structural representation of P5S-1H1 bound to the RBD (PDB ID: 7XS8), with the heavy and light chains shown in orange and red ribbons, respectively. The epitope footprint is displayed as blue surface, and hotspot residues involved in binding — Y421, Y453, L455, F456, and H505 — are highlighted in orange to indicate their energetic importance. (D) Close-up view of the RBD interface, emphasizing spatial localization of these hotspots and their role in stabilizing antibody engagement.(E) Detailed visualization of heavy chain interactions reveals that residues Y33, S53, F58, L99, and Y102 form critical contacts with the RBD. These residues are depicted as magenta sticks, annotated to illustrate their contributions to the binding interface.

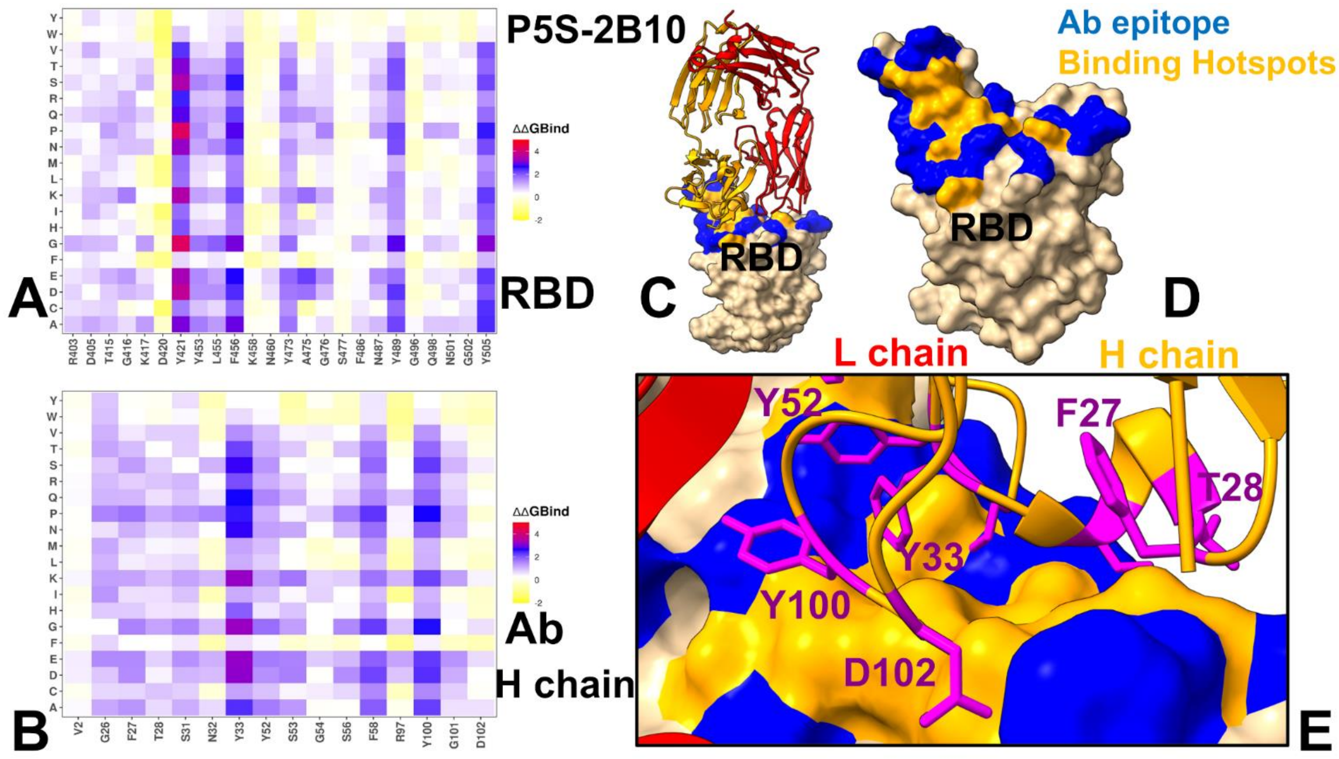

Figure S10. Mutational sensitivity and interfacial contacts in the P5S-2B10–RBD complex.
(A, B) Ensemble-based mutational scanning of the RBD and heavy chain residues contributing to P5S-2B10 binding interface. (C) Structural view of the P5S-2B10–RBD complex (PDB ID: 7XSC). The antibody's heavy and light chains are shown in orange and red ribbons, respectively. The epitope footprint is rendered in blue surface, with hotspot residues involved in binding — Y421, Y453, L455, F456, and H505 — highlighted in orange. (D) Close-up of the interface emphasizes the spatial organization of these hotspots and their overlap with ACE2-binding residues. (E) Detailed interaction map shows critical heavy chain contacts — Y33, F27, T28, Y52, Y100, D102 — as magenta sticks, illustrating their role in stabilizing the antibody–RBD interface.

Table S1. The list of Omicron Variants with Assigned Clade Annotation

clade	parent	Variant	WHO
19B	19A	9A: 19A	
20A	19A	19B: 19B	
20B	20A	20A: 20A	
20C	20A	20B: 20B	
20D	20B	20C: 20C	
20E	20A	20D: 20D	
20F	20B	20E: 20E	
20G	20C	20F: 20F	
20H	20C	20G: 20G	Beta
20I	20B	20I: 20I (Alpha)	Alpha
20J	20B	20J: 20J (Gamma)	Gamma
21A	20A	21A: 21A (Delta)	Delta
21B	20A	21B: 21B (Kappa)	Kappa
21C	20C	21C: 21C (Epsilon)	Epsilon
21D	20A	21D: 21D (Eta)	Eta
21E	20B	21E: 21E (Theta)	Theta
21F	20C	21F: 21F (Iota)	Iota
21G	20D	21G: 21G (Lambda)	Lambda
21H	20A	21I: 21I (Delta)	Mu
21I	21A	21H: 21H (Mu)	Delta
21J	21A	21J: 21J (Delta)	Delta
21K	21M	21K: 21K (BA.1)	Omicron
21L	21M	21L: 21L (BA.2)	Omicron
21M	20B	21M: 21M (Omicron)	Omicron
22A	21L	22A: 22A (BA.4)	Omicron
22B	21L	22B: 22B (BA.5)	Omicron
22C	21L	22C: 22C (BA.2.12.1)	Omicron
22D	21L	22D: 22D (BA.2.75)	Omicron
22E	22B	22E: 22E (BQ.1)	Omicron
22F	21L	22F: 22F (XBB)	Omicron
23A	22F	23A: 23A (XBB.1.5)	Omicron
23B	22F	23B: 23B (XBB.1.16)	Omicron
23C	22D	23C: 23C (CH.1.1)	Omicron
23D	22F	23D: 23D (XBB.1.9)	Omicron
23E	22F	23E: 23E (XBB.2.3)	Omicron
23F	23D	23F: 23F (EG.5.1)	Omicron
23G	23A	23G: 23G (XBB.1.5.70)	Omicron
23H	23F	23H: 23H (HK.3)	Omicron
23I	21L	23I: 23I (BA.2.86)	Omicron
24A	23I	24A: 24A (JN.1)	Omicron
24B	24A	24B: 24B (JN.1.11.1)	Omicron
24C	24B	24C: 24C (KP.3)	Omicron
24D	21L	24D: 24D (XDV.1)	Omicron

24E	24C	24E: 24E (KP.3.1.1)	Omicron
24F	24A	24F: 24F (XEC)	Omicron
24G	24B	24G: 24G (KP.2.3)	Omicron
24H	24A	24H: 24H (LF.7)	Omicron
24I	24A	24I: 24I (MV.1)	Omicron
25A	24B	25A: 25A (LP.8.1)	Omicron
25B	24D	25B: 25B (NB.1.8.1)	Omicron

Table S2. Mutational landscape of the Omicron variants.

Variant	Mutational landscape
BA.1	A67, T95I, G339D, S371L, S373P, S375F, K417N, N440K, G446S, S477N, T478K, E484A, Q493R, G496S, Q498R, N501Y, Y505H, T547K, D614G, H655Y, N679K, P681H, N764K, D796Y, N856K, Q954H, N969K, L981F
BA.2	T19I, G142D, V213G, G339D, S371F, S373P, S375F, T376A, D405N, R408S, K417N, N440K, S477N, T478K, E484A, Q493R, Q498R, N501Y, Y505H, D614G, H655Y, N679K, P681H, N764K, D796Y, Q954H, N969K
BA.4	T19I, G142D, V213G, G339D, S371F, S373P, S375F, T376A, D405N, R408S, K417N, N440K, L452R, S477N, T478K, E484A, F486V, R493Q reversal, Q498R, N501Y, Y505H, D614G, H655Y, N679K, P681H, N764K, D796Y, Q954H, N969K
BA.5	T19I, LPPA24-27S, Del 69-70, G142D, V213G, G339D, S371F, S373P, S375F, T376A, D405N, R408S, K417N, N440K, L452R, S477N, T478K, E484A, F486V, R493Q reversal, Q498R, N501Y, Y505H, D614G, H655Y, N679K, P681H, N764K, D796Y, Q954H, N969K
BQ.1.1	T19I, LPPA24-27S, H69del, V70del, V213G, G142D, G339D, S371F, S373P, S375F, T376A, D405N, R408S, K417N, N440K, K444T, L452R, N460K, S477N, T478K, E484A, F486V, R493Q reversal, Q498R, N501Y, Y505H, D614G, H655Y, N679K, P681H, N764K, D796Y, Q954H, N969K
XBB.1	T19I, V83A, G142D, Del144, H146Q, Q183E, V213E, G252V, G339H, R346T, L368I, S371F, S373P, S375F, T376A, D405N, R408S, K417N, N440K, V445P, G446S, N460K, S477N, T478K, E484A, **F486S, F490S**, R493Q reversal, Q498R, N501Y, Y505H, D614G, H655Y, N679K, P681H, N764K, D796Y, Q954H, N969K
XBB.1.5	T19I, V83A, G142D, Del144, H146Q, Q183E, V213E, G252V, G339H, R346T, L368I, S371F, S373P, S375F, T376A, D405N, R408S, K417N, N440K, V445P, G446S,N460K, S477N, T478K, E484A, **F486P, F490S** R493Q reversal, Q498R, N501Y, Y505H, D614G, H655Y, N679K, P681H, N764K, D796Y, Q954H, N969K
JN.1	T19I, R21T, S50L, del69-70,V127F, delY144, F157S, R158G, delN211, L213I, L226F, H25N, A264D, I332V, D339H, K356T, R403K, V445H, G446S, N450D, L452W, **L455S,** N460K, N481K, del V483, A484K, F486P, R493Q, E554K, A570V, P612S, I670V, H68R, D939F, P1143L
KP.2	**JN.1 + S:R346T, S:F456L, S:V1104L**<br>T19I, R21T, S50L, del69-70,V127F, delY144, F157S, R158G, delN211, L213I, L226F, H25N,A264D, I332V, D339H, **R346T**, K356T, R403K, V445H, G446S, N450D, L452W, **L455S, F456L**, N460K, N481K, del V483, A484K, F486P, R493Q, E554K, A570V, P612S, I670V, H68R, D939F, **V1104L,** P1143L
KP.3	**JN.1 + S:F456L, S:Q493E, S:V1104L**<br>T19I, R21T, S50L, del69-70,V127F, delY144, F157S, R158G, delN211, L213I, L226F, H25N,A264D, I332V, D339H, K356T, R403K, V445H, G446, N450D, L452W, **L455S, F456L**, N460K, N481K, del V483, A484K, F486P, **Q493E**, E554K, A570V, P612S, I670V, H68R, D939F, **V1104L,** P1143L

KP.1.1	**JN.1 + S:F456L, S:R346T, S:K1086R, S:V1104L**<br>T19I, R21T, S50L, del69-70,V127F, delY144, F157S, R158G, delN211, L213I, L226F, H25N,A264D, I332V, D339H, **R346T**, K356T, R403K, V445H, G446, N450D, L452W, **L455S, F456L**, N460K, N481K, del V483, A484K, F486P, **Q493E**, E554K, A570V, P612S, H68R, D939F, **K1086R**, **V1104L,** P1143L
LP.8	**KP.1.1+ F186L, H445R, Q493E, S31 del**<br>T19I, R21T, S31 del, S50L, del69-70,V127F, delY144, F157S, R158G, F186L, delN211, L213I, L226F, H25N,A264D, I332V, D339H, **R346T**, K356T, R403K, H445R, G446, N450D, L452W, **L455S, F456L**, N460K, N481K, del V483, A484K, F486P, **Q493E**, E554K, A570V, P612S, I670V, H68R, D939F, **K1086R**, **V1104L,** P1143L
LB.1	**JN.1+ S:S31-, S:Q183H, S:R346T, S:F456L**<br>T19I, R21T, **S31-**, S50L, del69-70,V127F, delY144, F157S, R158G, **Q183H**, delN211, L213I, L226F, H25N,A264D, I332V, D339H, **R346T,** K356T, R403K, V445H, G446S, N450D, L452W, **L455S, F456L**, N460K, N481K, del V483, A484K, F486P, R493Q, E554K, A570V, P612S, I670V, H68R, D939F, P1143L
XEC	**JN.1 + S:T22N, S:F59S, S:F456L, S:Q493E, S:V1104L**<br>T19I, R21T, **T22N**, S50L, **F59S**, del69-70,V127F, delY144, F157S, R158G, delN211, L213I, L226F, H25N,A264D, I332V, D339H, K356T, R403K, V445H, G446S, N450D, L452W, **L455S,** F456L. N460K, N481K, del V483, A484K, F486P, **Q493E**, E554K, A570V, P612S, I670V, H68R, D939F, **V1104L**, P1143L

Table S3. The list of the intermolecular contacts in the structure of the BD55-1205 complex with RBD (pdb id 8XE9).*

RBD Residue	RBD Residue Number	RBD chain	Ab Residue	Ab Residue Number	Ab chain
ARG	403	C	ASN	30	B
ARG	403	C	GLY	92	B
ASN	405	C	ASP	93	B
THR	415	C	SER	56	A
THR	415	C	THR	57	A
THR	415	C	PHE	58	A
GLY	416	C	TYR	52	A
GLY	416	C	SER	56	A
GLY	416	C	PHE	58	A
ASN	417	C	TYR	33	A
ASN	417	C	TYR	52	A
ASN	417	C	TRP	94	B
ASN	417	C	PRO	95	B
ASP	420	C	TYR	52	A
ASP	420	C	SER	56	A
TYR	421	C	TYR	33	A
TYR	421	C	TYR	52	A
TYR	421	C	PRO	53	A
TYR	421	C	GLY	54	A
TYR	421	C	GLY	55	A
TYR	453	C	ILE	101	A
LEU	455	C	TYR	33	A
LEU	455	C	PRO	53	A
LEU	455	C	TRP	94	B
LEU	455	C	LEU	99	A
LEU	455	C	ILE	101	A
LEU	455	C	ARG	102	A
PHE	456	C	ARG	31	A
PHE	456	C	ASN	32	A
PHE	456	C	TYR	33	A
PHE	456	C	PRO	53	A
PHE	456	C	LEU	99	A
ARG	457	C	PRO	53	A
ARG	457	C	GLY	54	A
LYS	458	C	SER	30	A
LYS	458	C	ARG	31	A
LYS	458	C	PRO	53	A
LYS	458	C	GLY	54	A
SER	459	C	PRO	53	A

SER	459	C	GLY	54	A
LYS	460	C	GLY	54	A
LYS	460	C	GLY	55	A
LYS	460	C	SER	56	A
TYR	473	C	SER	30	A
TYR	473	C	ARG	31	A
TYR	473	C	ASN	32	A
TYR	473	C	PRO	53	A
GLN	474	C	ARG	31	A
ALA	475	C	PHE	27	A
ALA	475	C	THR	28	A
ALA	475	C	ARG	31	A
ALA	475	C	ASN	32	A
ALA	475	C	ARG	97	A
GLY	476	C	GLY	26	A
GLY	476	C	PHE	27	A
GLY	476	C	THR	28	A
GLY	476	C	ARG	31	A
GLY	476	C	ASN	32	A
ASN	477	C	GLY	26	A
ASN	477	C	PHE	27	A
ASN	477	C	THR	28	A
PRO	486	C	GLU	104	A
ASN	487	C	VAL	2	A
ASN	487	C	GLY	26	A
ASN	487	C	PHE	27	A
ASN	487	C	ARG	97	A
ASN	487	C	GLU	104	A
TYR	489	C	ASN	32	A
TYR	489	C	ARG	97	A
TYR	489	C	LEU	99	A
TYR	489	C	ARG	102	A
TYR	489	C	GLU	104	A
SER	490	C	ARG	102	A
PRO	491	C	ARG	102	A
LEU	492	C	ARG	102	A
GLN	493	C	ILE	101	A
GLN	493	C	ARG	102	A
ARG	498	C	SER	31	B
ARG	498	C	SER	67	B
THR	500	C	SER	28	B
THR	500	C	PHE	29	B
THR	500	C	GLY	68	B

TYR	501	C	SER	28	B
TYR	501	C	PHE	29	B
TYR	501	C	ASN	30	B
TYR	501	C	SER	31	B
GLY	502	C	SER	28	B
GLY	502	C	PHE	29	B
GLY	502	C	ASN	30	B
VAL	503	C	SER	28	B
HIS	505	C	SER	28	B
HIS	505	C	PHE	29	B
HIS	505	C	ASN	30	B
HIS	505	C	GLY	92	B
HIS	505	C	ASP	93	B

*The total number of interfacial contacts is 95 which includes 2 charged-charged contacts; 14 charged-polar contacts; 22 charged-nonpolar contacts; 4 polar-polar contacts; 30 polar-nonpolar contacts; 23 nonpolar-nonpolar contacts.

Amino acids are classified into three categories based on their polarity: Charged: Arg, Asp, Glu), His, Lys. Polar: Asn, Gln, Ser, Thr. Nonpolar: Ala, Cys, Gly, Ile, Leu, Met, Phe, Pro, Trp, Tyr and Val. Interfacial Contacts are the contacts formed between amino acids at the interface of a protein complex within a specific distance threshold (e.g., 5.5 Å).

Table S4. The list of the intermolecular contacts in the structure of the BD-604 complex with RBD (pdb id 8HWT).*

RBD Residue	RBD Residue Number	RBD chain	Ab Residue	Ab Residue Number	Ab chain
ARG	403	A	SER	30	L
ARG	403	A	ASP	32	L
ASN	405	A	ASN	92	L
ASN	405	A	SER	93	L
GLU	406	A	SER	93	L
GLN	409	A	SER	93	L
THR	415	A	SER	56	H
THR	415	A	PHE	58	H
GLY	416	A	TYR	52	H
GLY	416	A	SER	56	H
GLY	416	A	PHE	58	H
ASN	417	A	TYR	33	H
ASN	417	A	TYR	52	H
ASP	420	A	TYR	52	H
ASP	420	A	SER	56	H
TYR	421	A	TYR	33	H
TYR	421	A	TYR	52	H
TYR	421	A	SER	53	H
TYR	421	A	GLY	54	H
TYR	453	A	PRO	101	H
LEU	455	A	TYR	33	H
LEU	455	A	LEU	99	H
LEU	455	A	GLY	100	H
LEU	455	A	PRO	101	H
LEU	455	A	TYR	102	H
PHE	456	A	SER	31	H
PHE	456	A	TYR	33	H
PHE	456	A	ASP	98	H
PHE	456	A	LEU	99	H
PHE	456	A	TYR	102	H
ARG	457	A	SER	53	H
LYS	458	A	SER	30	H
LYS	458	A	SER	31	H
LYS	458	A	SER	53	H
LYS	458	A	GLY	54	H

SER	459	A	SER	53	H
SER	459	A	GLY	54	H
ASN	460	A	SER	53	H
ASN	460	A	GLY	54	H
ASN	460	A	GLY	55	H
ASN	460	A	SER	56	H
TYR	473	A	SER	30	H
TYR	473	A	SER	31	H
TYR	473	A	ASN	32	H
TYR	473	A	SER	53	H
GLN	474	A	SER	31	H
GLN	474	A	ASN	32	H
ALA	475	A	GLY	26	H
ALA	475	A	ILE	27	H
ALA	475	A	ILE	28	H
ALA	475	A	SER	31	H
ALA	475	A	ASN	32	H
ALA	475	A	ARG	97	H
GLY	476	A	GLY	26	H
GLY	476	A	ILE	27	H
GLY	476	A	ILE	28	H
GLY	476	A	SER	31	H
GLY	476	A	ASN	32	H
ASN	477	A	SER	25	H
ASN	477	A	GLY	26	H
ASN	477	A	ILE	27	H
ASN	477	A	ILE	28	H
LYS	478	A	GLY	26	H
PHE	486	A	VAL	2	H
PHE	486	A	ARG	97	H
PHE	486	A	ASP	105	H
PHE	486	A	VAL	106	H
ASN	487	A	VAL	2	H
ASN	487	A	GLY	26	H
ASN	487	A	ILE	27	H
ASN	487	A	ARG	97	H
TYR	489	A	ARG	97	H
TYR	489	A	LEU	99	H
TYR	489	A	TYR	102	H
TYR	489	A	ASP	105	H
PHE	490	A	TYR	102	H
LEU	492	A	TYR	102	H
ARG	493	A	SER	31	L

ARG	493	A	ASP	32	L
ARG	493	A	ALA	50	L
ARG	493	A	PRO	101	H
ARG	493	A	TYR	102	H
ARG	498	A	SER	31	L
ARG	498	A	SER	67	L
THR	500	A	SER	67	L
THR	500	A	GLY	68	L
TYR	501	A	GLY	28	L
TYR	501	A	SER	30	L
TYR	501	A	SER	31	L
TYR	501	A	SER	67	L
TYR	501	A	GLY	68	L
GLY	502	A	GLN	27	L
GLY	502	A	GLY	28	L
GLY	502	A	ILE	29	L
GLY	502	A	SER	30	L
VAL	503	A	GLN	27	L
HIS	505	A	GLY	28	L
HIS	505	A	ILE	29	L
HIS	505	A	SER	30	L
HIS	505	A	ASP	32	L
HIS	505	A	ASN	92	L

*The total number of interfacial contacts is 101 which includes 3 charged-charged contacts; 13 charged-polar contacts; 14 charged-nonpolar contacts; 11 polar-polar contacts; 30 polar-nonpolar contacts; 30 nonpolar-nonpolar contacts.

Table S5. The list of the intermolecular contacts in the structure of the OMI-42 complex with RBD (pdb id 8CBF).*

RBD Residue	RBD Residue Number	RBD Chain	Ab Residue	Ab Residue Number	Ab Chain
ARG	403	E	GLU	52	L
ARG	403	E	LYS	55	L
ASP	405	E	ASN	33	L
GLU	406	E	TYR	34	L
ARG	408	E	GLY	30	L
ARG	408	E	GLY	31	L
GLN	409	E	TYR	32	L
GLN	409	E	TYR	34	L
GLN	414	E	TYR	32	L
THR	415	E	TYR	32	L
THR	415	E	TYR	34	L
THR	415	E	TYR	93	L
THR	415	E	GLY	95	L
THR	415	E	ASN	96	L
THR	415	E	TYR	109	H
GLY	416	E	TYR	32	L
GLY	416	E	TYR	34	L
GLY	416	E	TYR	93	L
GLY	416	E	TYR	109	H
LYS	417	E	TYR	34	L
LYS	417	E	GLU	52	L
LYS	417	E	LYS	55	L
LYS	417	E	TYR	109	H
LYS	417	E	TYR	110	H
ILE	418	E	TYR	34	L
ASP	420	E	TYR	93	L
ASP	420	E	SER	105	H
ASP	420	E	TYR	109	H
TYR	421	E	PRO	102	H
TYR	421	E	GLY	103	H
TYR	421	E	TYR	104	H
TYR	421	E	SER	105	H
TYR	421	E	SER	106	H
TYR	421	E	TYR	109	H
TYR	421	E	TYR	110	H
TYR	453	E	GLU	52	L
TYR	453	E	LYS	55	L
TYR	453	E	TYR	110	H
ARG	454	E	TYR	110	H

LEU	455	E	PHE	101	H
LEU	455	E	SER	106	H
LEU	455	E	TYR	110	H
PHE	456	E	PHE	101	H
PHE	456	E	PRO	102	H
PHE	456	E	SER	106	H
PHE	456	E	TYR	110	H
ARG	457	E	PRO	102	H
ARG	457	E	GLY	103	H
ARG	457	E	TYR	104	H
LYS	458	E	ASP	30	H
LYS	458	E	ASP	31	H
LYS	458	E	TRP	53	H
LYS	458	E	PRO	102	H
LYS	458	E	GLY	103	H
LYS	458	E	TYR	104	H
SER	459	E	TRP	53	H
ASN	460	E	TYR	104	H
TYR	473	E	PRO	28	H
TYR	473	E	ASP	31	H
TYR	473	E	TYR	32	H
TYR	473	E	PRO	102	H
GLN	474	E	PRO	28	H
ALA	475	E	VAL	2	H
ALA	475	E	GLY	26	H
ALA	475	E	PHE	27	H
ALA	475	E	PRO	28	H
ALA	475	E	TYR	32	H
ALA	475	E	LYS	98	H
ALA	475	E	ALA	100	H
GLY	476	E	VAL	2	H
GLY	476	E	GLY	26	H
GLY	476	E	PHE	27	H
GLY	476	E	PRO	28	H
SER	477	E	GLU	1	H
SER	477	E	GLY	26	H
SER	477	E	PHE	27	H
ASN	487	E	VAL	2	H
ASN	487	E	GLY	26	H
TYR	489	E	TYR	32	H
TYR	489	E	ALA	100	H
TYR	489	E	PHE	101	H
GLN	493	E	LYS	55	L

GLY	504	E	ASN	33	L
TYR	505	E	ASN	33	L
TYR	505	E	GLU	52	L
TYR	505	E	VAL	53	L
TYR	505	E	SER	54	L

*The total number of interfacial contacts is 87 which includes 6 charged-charged contacts; 4 charged-polar contacts; 21 charged-nonpolar contacts; 1 polar-polar contacts; 22 polar-nonpolar contacts; 33 nonpolar-nonpolar contacts.

Table S6. The list of the intermolecular contacts in the structure of the P5S-1H1 complex with RBD (pdb id 7XS8).

RBD Residue	RBD Residue Number	Residue Chain	Ab Residue	Ab Residue Number	Residue Chain
ARG	403	E	PHE	32	L
ASP	405	E	ASN	92	L
GLU	406	E	ASP	93	L
ARG	408	E	PHE	58	A
GLN	409	E	ASP	93	L
THR	415	E	PHE	58	A
THR	415	E	THR	57	A
THR	415	E	SER	56	A
GLY	416	E	TYR	52	A
GLY	416	E	SER	56	A
GLY	416	E	PHE	58	A
LYS	417	E	ASP	93	L
LYS	417	E	TYR	33	A
LYS	417	E	GLN	100	A
LYS	417	E	TYR	52	A
ASP	420	E	SER	56	A
ASP	420	E	PHE	58	A
ASP	420	E	TYR	52	A
TYR	421	E	TYR	52	A
TYR	421	E	SER	53	A
TYR	421	E	GLY	55	A
TYR	421	E	GLY	54	A
TYR	421	E	TYR	33	A
TYR	421	E	SER	56	A
TYR	449	E	ASN	31	L
TYR	453	E	VAL	101	A
TYR	453	E	PHE	32	L
ARG	454	E	TYR	33	A
LEU	455	E	GLN	100	A
LEU	455	E	VAL	101	A
LEU	455	E	TYR	33	A
LEU	455	E	LEU	99	A
PHE	456	E	TYR	33	A
PHE	456	E	TYR	102	A
PHE	456	E	LEU	99	A
PHE	456	E	ASP	98	A
PHE	456	E	GLN	100	A

PHE	456	E	ASN	32	A
ARG	457	E	SER	53	A
ARG	457	E	GLY	54	A
LYS	458	E	SER	31	A
LYS	458	E	GLY	54	A
LYS	458	E	SER	30	A
LYS	458	E	SER	53	A
SER	459	E	GLY	54	A
SER	459	E	SER	53	A
ASN	460	E	GLY	54	A
ASN	460	E	SER	56	A
ASN	460	E	GLY	55	A
ASN	460	E	SER	53	A
TYR	473	E	ASN	32	A
TYR	473	E	SER	30	A
TYR	473	E	SER	53	A
TYR	473	E	SER	31	A
GLN	474	E	SER	31	A
ALA	475	E	SER	31	A
ALA	475	E	GLY	26	A
ALA	475	E	ILE	27	A
ALA	475	E	THR	28	A
ALA	475	E	ASN	32	A
ALA	475	E	ARG	97	A
GLY	476	E	THR	28	A
GLY	476	E	ASN	32	A
GLY	476	E	SER	31	A
GLY	476	E	GLY	26	A
GLY	476	E	ILE	27	A
SER	477	E	GLY	26	A
SER	477	E	THR	28	A
GLU	484	E	TYR	102	A
PHE	486	E	GLY	26	A
PHE	486	E	ASP	105	A
PHE	486	E	VAL	106	A
PHE	486	E	ARG	97	A
PHE	486	E	ILE	27	A
PHE	486	E	VAL	2	A
ASN	487	E	GLY	26	A
ASN	487	E	LEU	99	A
ASN	487	E	ILE	27	A
ASN	487	E	ASP	105	A
ASN	487	E	THR	28	A

ASN	487	E	ASN	32	A
ASN	487	E	ARG	97	A
TYR	489	E	ARG	97	A
TYR	489	E	TYR	102	A
TYR	489	E	LEU	99	A
TYR	489	E	ASN	32	A
TYR	489	E	ASP	105	A
PHE	490	E	TYR	102	A
GLN	493	E	VAL	101	A
GLN	493	E	TYR	102	A
SER	494	E	PHE	32	L
TYR	495	E	PHE	32	L
TYR	495	E	SER	30	L
GLY	496	E	PHE	32	L
GLY	496	E	SER	30	L
PHE	497	E	SER	30	L
GLN	498	E	SER	30	L
GLN	498	E	GLY	68	L
GLN	498	E	SER	67	L
GLN	498	E	ASN	31	L
THR	500	E	SER	67	L
THR	500	E	THR	69	L
THR	500	E	ILE	29	L
THR	500	E	GLN	27	L
THR	500	E	GLY	28	L
THR	500	E	GLY	68	L
ASN	501	E	GLY	68	L
ASN	501	E	ILE	29	L
ASN	501	E	SER	30	L
ASN	501	E	GLY	28	L
GLY	502	E	GLN	27	L
GLY	502	E	GLY	28	L
GLY	502	E	ILE	29	L
VAL	503	E	GLN	27	L
GLY	504	E	GLN	27	L
TYR	505	E	HIS	90	L
TYR	505	E	ILE	2	L
TYR	505	E	ASN	92	L
TYR	505	E	GLN	27	L
TYR	505	E	GLY	28	L
TYR	505	E	ILE	29	L

*The total number of interfacial contacts is 121 which includes 2 charged-charged contacts; 10 charged-polar contacts; 17 charged-nonpolar contacts; 16 polar-polar contacts; 44 polar-nonpolar contacts; 32 nonpolar-nonpolar contacts.

Table S7. The list of the intermolecular contacts in the structure of the P5S-2B10 complex with RBD (pdb id 7XSC).

RBD Residue	RBD Residue Number	RBD Chain	Ab Residue	AB Residue Number	AB Chain
ARG	403	E	ASN	93	B
ARG	403	E	ASP	92	B
ASP	405	E	GLN	27	B
ASP	405	E	ASN	93	B
THR	415	E	THR	57	A
THR	415	E	SER	56	A
THR	415	E	PHE	58	A
GLY	416	E	TYR	52	A
GLY	416	E	PHE	58	A
GLY	416	E	SER	56	A
LYS	417	E	ASP	92	B
LYS	417	E	TYR	33	A
LYS	417	E	TYR	52	A
LYS	417	E	TYR	100	A
ASP	420	E	PHE	58	A
ASP	420	E	TYR	52	A
ASP	420	E	SER	56	A
TYR	421	E	SER	56	A
TYR	421	E	SER	53	A
TYR	421	E	TYR	33	A
TYR	421	E	TYR	52	A
TYR	421	E	GLY	54	A
TYR	421	E	GLY	55	A
TYR	453	E	ASP	92	B
TYR	453	E	PHE	32	B
LEU	455	E	SER	53	A
LEU	455	E	TYR	33	A
LEU	455	E	TYR	100	A
PHE	456	E	SER	53	A
PHE	456	E	TYR	33	A
PHE	456	E	SER	31	A
PHE	456	E	TYR	100	A
PHE	456	E	ASN	32	A
PHE	456	E	GLY	101	A
ARG	457	E	SER	53	A

ARG	457	E	GLY	54	A
LYS	458	E	ARG	71	A
LYS	458	E	SER	31	A
LYS	458	E	SER	53	A
LYS	458	E	SER	30	A
LYS	458	E	GLY	54	A
LYS	458	E	GLY	55	A
SER	459	E	GLY	54	A
SER	459	E	SER	53	A
ASN	460	E	SER	56	A
ASN	460	E	GLY	54	A
ASN	460	E	GLY	55	A
TYR	473	E	ASN	32	A
TYR	473	E	SER	31	A
TYR	473	E	SER	53	A
TYR	473	E	SER	30	A
GLN	474	E	SER	31	A
ALA	475	E	PHE	27	A
ALA	475	E	ARG	97	A
ALA	475	E	THR	28	A
ALA	475	E	ASN	32	A
ALA	475	E	SER	31	A
GLY	476	E	ASN	32	A
GLY	476	E	GLY	26	A
GLY	476	E	PHE	27	A
GLY	476	E	THR	28	A
SER	477	E	THR	28	A
SER	477	E	PHE	27	A
GLY	485	E	ASP	102	A
PHE	486	E	VAL	2	A
PHE	486	E	GLY	26	A
PHE	486	E	ASP	102	A
PHE	486	E	ARG	97	A
ASN	487	E	ASN	32	A
ASN	487	E	THR	28	A
ASN	487	E	GLY	26	A
ASN	487	E	ASP	102	A
ASN	487	E	PHE	27	A
ASN	487	E	ARG	97	A
CYS	488	E	ASP	102	A
TYR	489	E	ASN	32	A
TYR	489	E	ASP	102	A
TYR	489	E	ARG	97	A

TYR	489	E	GLY	101	A
GLY	496	E	ARG	30	B
GLN	498	E	ARG	30	B
THR	500	E	ARG	30	B
THR	500	E	ASP	28	B
ASN	501	E	ARG	30	B
ASN	501	E	ASP	28	B
GLY	502	E	ARG	30	B
GLY	502	E	ASP	28	B
TYR	505	E	ASN	93	B
TYR	505	E	PHE	32	B
TYR	505	E	ILE	29	B
TYR	505	E	ARG	30	B
TYR	505	E	ASP	28	B
TYR	505	E	ASP	92	B

*The total number of interfacial contacts is 103 which includes 3 charged-charged contacts; 15 charged-polar contacts; 22 charged-nonpolar contacts; 8 polar-polar contacts; 25 polar-nonpolar contacts; 20 nonpolar-nonpolar contacts.
