# Supplementary material for: Multiscale Modeling and Dynamic Mutational Profiling of Binding Energetics and Immune Escape for Class I Antibodies with SARS-CoV-2 Spike Protein: Dissecting Mechanisms of High Resistance to Viral Escape Against Emerging Variants
Source: Viruses. 2025 Jul 23;17(8):1029. doi: 10.3390/v17081029 (PMC12390076; doi:10.3390/v17081029)
Supplement: Supplementary file 1 [file viruses-17-01029-s001.zip › viruses-3717688-supplementary/SUPPLEMENTARY MATERIALS/Table S1.pdf]

**Table S1. The list of Omicron Variants with Assigned Clade Annotation**

| clade | parent | Variant | WHO |
|---|---|---|---|
| 19B | 19A | 9A: 19A | |
| 20A | 19A | 19B: 19B | |
| 20B | 20A | 20A: 20A | |
| 20C | 20A | 20B: 20B | |
| 20D | 20B | 20C: 20C | |
| 20E | 20A | 20D: 20D | |
| 20F | 20B | 20E: 20E | |
| 20G | 20C | 20F: 20F | |
| 20H | 20C | 20G: 20G | Beta |
| 20I | 20B | 20I: 20I (Alpha) | Alpha |
| 20J | 20B | 20J: 20J (Gamma) | Gamma |
| 21A | 20A | 21A: 21A (Delta) | Delta |
| 21B | 20A | 21B: 21B (Kappa) | Kappa |
| 21C | 20C | 21C: 21C (Epsilon) | Epsilon |
| 21D | 20A | 21D: 21D (Eta) | Eta |
| 21E | 20B | 21E: 21E (Theta) | Theta |
| 21F | 20C | 21F: 21F (Iota) | Iota |
| 21G | 20D | 21G: 21G (Lambda) | Lambda |
| 21H | 20A | 21I: 21I (Delta) | Mu |
| 21I | 21A | 21H: 21H (Mu) | Delta |
| 21J | 21A | 21J: 21J (Delta) | Delta |
| 21K | 21M | 21K: 21K (BA.1) | Omicron |
| 21L | 21M | 21L: 21L (BA.2) | Omicron |
| 21M | 20B | 21M: 21M (Omicron) | Omicron |

| | | | |
|---|---|---|---|
| 22A | 21L | 22A: 22A (BA.4) | Omicron |
| 22B | 21L | 22B: 22B (BA.5) | Omicron |
| 22C | 21L | 22C: 22C (BA.2.12.1) | Omicron |
| 22D | 21L | 22D: 22D (BA.2.75) | Omicron |
| 22E | 22B | 22E: 22E (BQ.1) | Omicron |
| 22F | 21L | 22F: 22F (XBB) | Omicron |
| 23A | 22F | 23A: 23A (XBB.1.5) | Omicron |
| 23B | 22F | 23B: 23B (XBB.1.16) | Omicron |
| 23C | 22D | 23C: 23C (CH.1.1) | Omicron |
| 23D | 22F | 23D: 23D (XBB.1.9) | Omicron |
| 23E | 22F | 23E: 23E (XBB.2.3) | Omicron |
| 23F | 23D | 23F: 23F (EG.5.1) | Omicron |
| 23G | 23A | 23G: 23G (XBB.1.5.70) | Omicron |
| 23H | 23F | 23H: 23H (HK.3) | Omicron |
| 23I | 21L | 23I: 23I (BA.2.86) | Omicron |
| 24A | 23I | 24A: 24A (JN.1) | Omicron |
| 24B | 24A | 24B: 24B (JN.1.11.1) | Omicron |
| 24C | 24B | 24C: 24C (KP.3) | Omicron |
| 24D | 21L | 24D: 24D (XDV.1) | Omicron |
| 24E | 24C | 24E: 24E (KP.3.1.1) | Omicron |
| 24F | 24A | 24F: 24F (XEC) | Omicron |
| 24G | 24B | 24G: 24G (KP.2.3) | Omicron |
| 24H | 24A | 24H: 24H (LF.7) | Omicron |
| 24I | 24A | 24I: 24I (MV.1) | Omicron |
| 25A | 24B | 25A: 25A (LP.8.1) | Omicron |
| 25B | 24D | 25B: 25B (NB.1.8.1) | Omicron |
