# Supplementary material for: Multiscale Modeling and Dynamic Mutational Profiling of Binding Energetics and Immune Escape for Class I Antibodies with SARS-CoV-2 Spike Protein: Dissecting Mechanisms of High Resistance to Viral Escape Against Emerging Variants
Source: Viruses. 2025 Jul 23;17(8):1029. doi: 10.3390/v17081029 (PMC12390076; doi:10.3390/v17081029)
Supplement: Supplementary file 1 [file viruses-17-01029-s001.zip › viruses-3717688-supplementary/SUPPLEMENTARY MATERIALS/Table S2.pdf]

Table S2. Mutational landscape of the Omicron variants.

**Variant**	**Mutational landscape**
BA.1	A67, T95I, G339D, S371L, S373P, S375F, K417N, N440K, G446S, S477N, T478K, E484A, Q493R, G496S, Q498R, N501Y, Y505H, T547K, D614G, H655Y, N679K, P681H, N764K, D796Y, N856K, Q954H, N969K, L981F
BA.2	T19I, G142D, V213G, G339D, S371F, S373P, S375F, T376A, D405N, R408S, K417N, N440K, S477N, T478K, E484A, Q493R, Q498R, N501Y, Y505H, D614G, H655Y, N679K, P681H, N764K, D796Y, Q954H, N969K
BA.4	T19I, G142D, V213G, G339D, S371F, S373P, S375F, T376A, D405N, R408S, K417N, N440K, L452R, S477N, T478K, E484A, F486V, R493Q reversal, Q498R, N501Y, Y505H, D614G, H655Y, N679K, P681H, N764K, D796Y, Q954H, N969K
BA.5	T19I, LPPA24-27S, Del 69-70, G142D, V213G, G339D, S371F, S373P, S375F, T376A, D405N, R408S, K417N, N440K, L452R, S477N, T478K, E484A, F486V, R493Q reversal, Q498R, N501Y, Y505H, D614G, H655Y, N679K, P681H, N764K, D796Y, Q954H, N969K
BQ.1.1	T19I, LPPA24-27S, H69del, V70del, V213G, G142D, G339D, S371F, S373P, S375F, T376A, D405N, R408S, K417N, N440K, K444T, L452R, N460K, S477N, T478K, E484A, F486V, R493Q reversal, Q498R, N501Y, Y505H, D614G, H655Y, N679K, P681H, N764K, D796Y, Q954H, N969K
XBB.1	T19I, V83A, G142D, Del144, H146Q, Q183E, V213E, G252V, G339H, R346T, L368I, S371F, S373P, S375F, T376A, D405N, R408S, K417N, N440K, V445P, G446S, N460K, S477N, T478K, E484A, **F486S, F490S**, R493Q reversal, Q498R, N501Y, Y505H, D614G, H655Y, N679K, P681H, N764K, D796Y, Q954H, N969K
XBB.1.5	T19I, V83A, G142D, Del144, H146Q, Q183E, V213E, G252V, G339H, R346T, L368I, S371F, S373P, S375F, T376A, D405N, R408S, K417N, N440K, V445P, G446S,N460K, S477N, T478K, E484A, **F486P, F490S**, R493Q reversal, Q498R, N501Y, Y505H, D614G, H655Y, N679K, P681H, N764K, D796Y, Q954H, N969K
JN.1	T19I, R21T, S50L, del69-70,V127F, delY144, F157S, R158G, delN211, L213I, L226F, H25N, A264D, I332V, D339H, K356T, R403K, V445H, G446S, N450D, L452W, **L455S,** N460K, N481K, del V483, A484K, F486P, R493Q, E554K, A570V, P612S, I670V, H68R, D939F, P1143L
KP.2	**JN.1 + S:R346T, S:F456L, S:V1104L** T19I, R21T, S50L, del69-70,V127F, delY144, F157S, R158G, delN211, L213I, L226F, H25N,A264D, I332V, D339H, **R346T**, K356T, R403K, V445H, G446S, N450D, L452W,

	**L455S, F456L**, N460K, N481K, del V483, A484K, F486P, R493Q, E554K, A570V, P612S, I670V, H68R, D939F, **V1104L,** P1143L
KP.3	**JN.1 + S:F456L, S:Q493E, S:V1104L**<br><br>T19I, R21T, S50L, del69-70,V127F, delY144, F157S, R158G, delN211, L213I, L226F, H25N,A264D, I332V, D339H, K356T, R403K, V445H, G446, N450D, L452W, **L455S, F456L**, N460K, N481K, del V483, A484K, F486P, **Q493E**, E554K, A570V, P612S, I670V, H68R, D939F, **V1104L,** P1143L
KP.1.1	**JN.1 + S:F456L, S:R346T, S:K1086R, S:V1104L**<br><br>T19I, R21T, S50L, del69-70,V127F, delY144, F157S, R158G, delN211, L213I, L226F, H25N,A264D, I332V, D339H, **R346T**, K356T, R403K, V445H, G446, N450D, L452W, **L455S, F456L**, N460K, N481K, del V483, A484K, F486P, **Q493E**, E554K, A570V, P612S, H68R, D939F, **K1086R**, **V1104L,** P1143L
LP.8	**KP.1.1+ F186L, H445R, Q493E, S31 del**<br><br>T19I, R21T, S31 del, S50L, del69-70,V127F, delY144, F157S, R158G, F186L, delN211, L213I, L226F, H25N,A264D, I332V, D339H, **R346T**, K356T, R403K, H445R, G446, N450D, L452W, **L455S, F456L**, N460K, N481K, del V483, A484K, F486P, **Q493E**, E554K, A570V, P612S, I670V, H68R, D939F, **K1086R**, **V1104L,** P1143L
LB.1	**JN.1+ S:S31-, S:Q183H, S:R346T, S:F456L**<br><br>T19I, R21T, **S31-**, S50L, del69-70,V127F, delY144, F157S, R158G, **Q183H**, delN211, L213I, L226F, H25N,A264D, I332V, D339H, **R346T,** K356T, R403K, V445H, G446S, N450D, L452W, **L455S, F456L**, N460K, N481K, del V483, A484K, F486P, R493Q, E554K, A570V, P612S, I670V, H68R, D939F, P1143L
XEC	**JN.1 + S:T22N, S:F59S, S:F456L, S:Q493E, S:V1104L**<br><br>T19I, R21T, **T22N**, S50L, **F59S**, del69-70,V127F, delY144, F157S, R158G, delN211, L213I, L226F, H25N,A264D, I332V, D339H, K356T, R403K, V445H, G446S, N450D, L452W, **L455S,** F456L. N460K, N481K, del V483, A484K, F486P, **Q493E**, E554K, A570V, P612S, I670V, H68R, D939F, **V1104L**, P1143L
