# Supplementary material for: Multiscale Modeling and Dynamic Mutational Profiling of Binding Energetics and Immune Escape for Class I Antibodies with SARS-CoV-2 Spike Protein: Dissecting Mechanisms of High Resistance to Viral Escape Against Emerging Variants
Source: Viruses. 2025 Jul 23;17(8):1029. doi: 10.3390/v17081029 (PMC12390076; doi:10.3390/v17081029)
Supplement: Supplementary file 1 [file viruses-17-01029-s001.zip › viruses-3717688-supplementary/SUPPLEMENTARY MATERIALS/Table S3.pdf]

Table S3. The list of the intermolecular contacts in the structure of the BD55-1205 complex with RBD (pdb id 8XE9).*

**RBD Residue**	**RBD Residue Number**	**RBD chain**	**Ab Residue**	**Ab Residue Number**	**Ab chain**
ARG	403	C	ASN	30	B
ARG	403	C	GLY	92	B
ASN	405	C	ASP	93	B
THR	415	C	SER	56	A
THR	415	C	THR	57	A
THR	415	C	PHE	58	A
GLY	416	C	TYR	52	A
GLY	416	C	SER	56	A
GLY	416	C	PHE	58	A
ASN	417	C	TYR	33	A
ASN	417	C	TYR	52	A
ASN	417	C	TRP	94	B
ASN	417	C	PRO	95	B
ASP	420	C	TYR	52	A
ASP	420	C	SER	56	A
TYR	421	C	TYR	33	A
TYR	421	C	TYR	52	A
TYR	421	C	PRO	53	A
TYR	421	C	GLY	54	A
TYR	421	C	GLY	55	A
TYR	453	C	ILE	101	A
LEU	455	C	TYR	33	A
LEU	455	C	PRO	53	A

LEU	455	C	TRP	94	B
LEU	455	C	LEU	99	A
LEU	455	C	ILE	101	A
LEU	455	C	ARG	102	A
PHE	456	C	ARG	31	A
PHE	456	C	ASN	32	A
PHE	456	C	TYR	33	A
PHE	456	C	PRO	53	A
PHE	456	C	LEU	99	A
ARG	457	C	PRO	53	A
ARG	457	C	GLY	54	A
LYS	458	C	SER	30	A
LYS	458	C	ARG	31	A
LYS	458	C	PRO	53	A
LYS	458	C	GLY	54	A
SER	459	C	PRO	53	A
SER	459	C	GLY	54	A
LYS	460	C	GLY	54	A
LYS	460	C	GLY	55	A
LYS	460	C	SER	56	A
TYR	473	C	SER	30	A
TYR	473	C	ARG	31	A
TYR	473	C	ASN	32	A
TYR	473	C	PRO	53	A
GLN	474	C	ARG	31	A
ALA	475	C	PHE	27	A

ALA	475	C	THR	28	A
ALA	475	C	ARG	31	A
ALA	475	C	ASN	32	A
ALA	475	C	ARG	97	A
GLY	476	C	GLY	26	A
GLY	476	C	PHE	27	A
GLY	476	C	THR	28	A
GLY	476	C	ARG	31	A
GLY	476	C	ASN	32	A
ASN	477	C	GLY	26	A
ASN	477	C	PHE	27	A
ASN	477	C	THR	28	A
PRO	486	C	GLU	104	A
ASN	487	C	VAL	2	A
ASN	487	C	GLY	26	A
ASN	487	C	PHE	27	A
ASN	487	C	ARG	97	A
ASN	487	C	GLU	104	A
TYR	489	C	ASN	32	A
TYR	489	C	ARG	97	A
TYR	489	C	LEU	99	A
TYR	489	C	ARG	102	A
TYR	489	C	GLU	104	A
SER	490	C	ARG	102	A
PRO	491	C	ARG	102	A
LEU	492	C	ARG	102	A

GLN	493	C	ILE	101	A
GLN	493	C	ARG	102	A
ARG	498	C	SER	31	B
ARG	498	C	SER	67	B
THR	500	C	SER	28	B
THR	500	C	PHE	29	B
THR	500	C	GLY	68	B
TYR	501	C	SER	28	B
TYR	501	C	PHE	29	B
TYR	501	C	ASN	30	B
TYR	501	C	SER	31	B
GLY	502	C	SER	28	B
GLY	502	C	PHE	29	B
GLY	502	C	ASN	30	B
VAL	503	C	SER	28	B
HIS	505	C	SER	28	B
HIS	505	C	PHE	29	B
HIS	505	C	ASN	30	B
HIS	505	C	GLY	92	B
HIS	505	C	ASP	93	B

*The total number of interfacial contacts is 95 which includes 2 charged-charged contacts; 14 charged-polar contacts; 22 charged-nonpolar contacts; 4 polar-polar contacts; 30 polar-nonpolar contacts; 23 nonpolar-nonpolar contacts.

Amino acids are classified into three categories based on their polarity: Charged: Arg, Asp, Glu), His, Lys. Polar: Asn, Gln, Ser, Thr. Nonpolar: Ala, Cys, Gly, Ile, Leu, Met, Phe, Pro, Trp, Tyr and Val. Interfacial Contacts are the contacts formed between amino acids at the interface of a protein complex within a specific distance threshold (e.g., 5.5 Å).
