# Supplementary material for: Multiscale Modeling and Dynamic Mutational Profiling of Binding Energetics and Immune Escape for Class I Antibodies with SARS-CoV-2 Spike Protein: Dissecting Mechanisms of High Resistance to Viral Escape Against Emerging Variants
Source: Viruses. 2025 Jul 23;17(8):1029. doi: 10.3390/v17081029 (PMC12390076; doi:10.3390/v17081029)
Supplement: Supplementary file 1 [file viruses-17-01029-s001.zip › viruses-3717688-supplementary/SUPPLEMENTARY MATERIALS/Table S4.pdf]

Table S4. The list of the intermolecular contacts in the structure of the BD-604 complex with RBD (pdb id 8HWT).*

RBD Residue	RBD Residue Number	RBD chain	Ab Residue	Ab Residue Number	Ab chain
ARG	403	A	SER	30	L
ARG	403	A	ASP	32	L
ASN	405	A	ASN	92	L
ASN	405	A	SER	93	L
GLU	406	A	SER	93	L
GLN	409	A	SER	93	L
THR	415	A	SER	56	H
THR	415	A	PHE	58	H
GLY	416	A	TYR	52	H
GLY	416	A	SER	56	H
GLY	416	A	PHE	58	H
ASN	417	A	TYR	33	H
ASN	417	A	TYR	52	H
ASP	420	A	TYR	52	H
ASP	420	A	SER	56	H
TYR	421	A	TYR	33	H
TYR	421	A	TYR	52	H
TYR	421	A	SER	53	H
TYR	421	A	GLY	54	H
TYR	453	A	PRO	101	H
LEU	455	A	TYR	33	H

LEU	455	A	LEU	99	H
LEU	455	A	GLY	100	H
LEU	455	A	PRO	101	H
LEU	455	A	TYR	102	H
PHE	456	A	SER	31	H
PHE	456	A	TYR	33	H
PHE	456	A	ASP	98	H
PHE	456	A	LEU	99	H
PHE	456	A	TYR	102	H
ARG	457	A	SER	53	H
LYS	458	A	SER	30	H
LYS	458	A	SER	31	H
LYS	458	A	SER	53	H
LYS	458	A	GLY	54	H
SER	459	A	SER	53	H
SER	459	A	GLY	54	H
ASN	460	A	SER	53	H
ASN	460	A	GLY	54	H
ASN	460	A	GLY	55	H
ASN	460	A	SER	56	H
TYR	473	A	SER	30	H
TYR	473	A	SER	31	H
TYR	473	A	ASN	32	H
TYR	473	A	SER	53	H
GLN	474	A	SER	31	H
GLN	474	A	ASN	32	H

ALA	475	A	GLY	26	H
ALA	475	A	ILE	27	H
ALA	475	A	ILE	28	H
ALA	475	A	SER	31	H
ALA	475	A	ASN	32	H
ALA	475	A	ARG	97	H
GLY	476	A	GLY	26	H
GLY	476	A	ILE	27	H
GLY	476	A	ILE	28	H
GLY	476	A	SER	31	H
GLY	476	A	ASN	32	H
ASN	477	A	SER	25	H
ASN	477	A	GLY	26	H
ASN	477	A	ILE	27	H
ASN	477	A	ILE	28	H
LYS	478	A	GLY	26	H
PHE	486	A	VAL	2	H
PHE	486	A	ARG	97	H
PHE	486	A	ASP	105	H
PHE	486	A	VAL	106	H
ASN	487	A	VAL	2	H
ASN	487	A	GLY	26	H
ASN	487	A	ILE	27	H
ASN	487	A	ARG	97	H
TYR	489	A	ARG	97	H
TYR	489	A	LEU	99	H

TYR	489	A	TYR	102	H
TYR	489	A	ASP	105	H
PHE	490	A	TYR	102	H
LEU	492	A	TYR	102	H
ARG	493	A	SER	31	L
ARG	493	A	ASP	32	L
ARG	493	A	ALA	50	L
ARG	493	A	PRO	101	H
ARG	493	A	TYR	102	H
ARG	498	A	SER	31	L
ARG	498	A	SER	67	L
THR	500	A	SER	67	L
THR	500	A	GLY	68	L
TYR	501	A	GLY	28	L
TYR	501	A	SER	30	L
TYR	501	A	SER	31	L
TYR	501	A	SER	67	L
TYR	501	A	GLY	68	L
GLY	502	A	GLN	27	L
GLY	502	A	GLY	28	L
GLY	502	A	ILE	29	L
GLY	502	A	SER	30	L
VAL	503	A	GLN	27	L
HIS	505	A	GLY	28	L
HIS	505	A	ILE	29	L
HIS	505	A	SER	30	L

HIS	505	A	ASP	32	L
HIS	505	A	ASN	92	L

*The total number of interfacial contacts is 101 which includes 3 charged-charged contacts; 13 charged-polar contacts; 14 charged-nonpolar contacts; 11 polar-polar contacts; 30 polar-nonpolar contacts; 30 nonpolar-nonpolar contacts.
