# Supplementary material for: Multiscale Modeling and Dynamic Mutational Profiling of Binding Energetics and Immune Escape for Class I Antibodies with SARS-CoV-2 Spike Protein: Dissecting Mechanisms of High Resistance to Viral Escape Against Emerging Variants
Source: Viruses. 2025 Jul 23;17(8):1029. doi: 10.3390/v17081029 (PMC12390076; doi:10.3390/v17081029)
Supplement: Supplementary file 1 [file viruses-17-01029-s001.zip › viruses-3717688-supplementary/SUPPLEMENTARY MATERIALS/Table S5.pdf]

Table S5. The list of the intermolecular contacts in the structure of the OMI-42 complex with RBD (pdb id 8CBF).*

RBD Residue	RBD Residue Number	RBD Chain	Ab Residue	Ab Residue Number	Ab Chain
ARG	403	E	GLU	52	L
ARG	403	E	LYS	55	L
ASP	405	E	ASN	33	L
GLU	406	E	TYR	34	L
ARG	408	E	GLY	30	L
ARG	408	E	GLY	31	L
GLN	409	E	TYR	32	L
GLN	409	E	TYR	34	L
GLN	414	E	TYR	32	L
THR	415	E	TYR	32	L
THR	415	E	TYR	34	L
THR	415	E	TYR	93	L
THR	415	E	GLY	95	L
THR	415	E	ASN	96	L
THR	415	E	TYR	109	H
GLY	416	E	TYR	32	L
GLY	416	E	TYR	34	L
GLY	416	E	TYR	93	L
GLY	416	E	TYR	109	H
LYS	417	E	TYR	34	L
LYS	417	E	GLU	52	L
LYS	417	E	LYS	55	L
LYS	417	E	TYR	109	H

LYS	417	E	TYR	110	H
ILE	418	E	TYR	34	L
ASP	420	E	TYR	93	L
ASP	420	E	SER	105	H
ASP	420	E	TYR	109	H
TYR	421	E	PRO	102	H
TYR	421	E	GLY	103	H
TYR	421	E	TYR	104	H
TYR	421	E	SER	105	H
TYR	421	E	SER	106	H
TYR	421	E	TYR	109	H
TYR	421	E	TYR	110	H
TYR	453	E	GLU	52	L
TYR	453	E	LYS	55	L
TYR	453	E	TYR	110	H
ARG	454	E	TYR	110	H
LEU	455	E	PHE	101	H
LEU	455	E	SER	106	H
LEU	455	E	TYR	110	H
PHE	456	E	PHE	101	H
PHE	456	E	PRO	102	H
PHE	456	E	SER	106	H
PHE	456	E	TYR	110	H
ARG	457	E	PRO	102	H
ARG	457	E	GLY	103	H
ARG	457	E	TYR	104	H

LYS	458	E	ASP	30	H
LYS	458	E	ASP	31	H
LYS	458	E	TRP	53	H
LYS	458	E	PRO	102	H
LYS	458	E	GLY	103	H
LYS	458	E	TYR	104	H
SER	459	E	TRP	53	H
ASN	460	E	TYR	104	H
TYR	473	E	PRO	28	H
TYR	473	E	ASP	31	H
TYR	473	E	TYR	32	H
TYR	473	E	PRO	102	H
GLN	474	E	PRO	28	H
ALA	475	E	VAL	2	H
ALA	475	E	GLY	26	H
ALA	475	E	PHE	27	H
ALA	475	E	PRO	28	H
ALA	475	E	TYR	32	H
ALA	475	E	LYS	98	H
ALA	475	E	ALA	100	H
GLY	476	E	VAL	2	H
GLY	476	E	GLY	26	H
GLY	476	E	PHE	27	H
GLY	476	E	PRO	28	H
SER	477	E	GLU	1	H
SER	477	E	GLY	26	H

SER	477	E	PHE	27	H
ASN	487	E	VAL	2	H
ASN	487	E	GLY	26	H
TYR	489	E	TYR	32	H
TYR	489	E	ALA	100	H
TYR	489	E	PHE	101	H
GLN	493	E	LYS	55	L
GLY	504	E	ASN	33	L
TYR	505	E	ASN	33	L
TYR	505	E	GLU	52	L
TYR	505	E	VAL	53	L
TYR	505	E	SER	54	L

*The total number of interfacial contacts is 87 which includes 6 charged-charged contacts; 4 charged-polar contacts; 21 charged-nonpolar contacts; 1 polar-polar contacts; 22 polar-nonpolar contacts; 33 nonpolar-nonpolar contacts.
