# Supplementary material for: Multiscale Modeling and Dynamic Mutational Profiling of Binding Energetics and Immune Escape for Class I Antibodies with SARS-CoV-2 Spike Protein: Dissecting Mechanisms of High Resistance to Viral Escape Against Emerging Variants
Source: Viruses. 2025 Jul 23;17(8):1029. doi: 10.3390/v17081029 (PMC12390076; doi:10.3390/v17081029)
Supplement: Supplementary file 1 [file viruses-17-01029-s001.zip › viruses-3717688-supplementary/SUPPLEMENTARY MATERIALS/Table S6.pdf]

Table S6. The list of the intermolecular contacts in the structure of the P5S-1H1 complex with RBD (pdb id 7XS8).

RBD Residue	RBD Residue Number	Residue Chain	Ab Residue	Ab Residue Number	Residue Chain
ARG	403	E	PHE	32	L
ASP	405	E	ASN	92	L
GLU	406	E	ASP	93	L
ARG	408	E	PHE	58	A
GLN	409	E	ASP	93	L
THR	415	E	PHE	58	A
THR	415	E	THR	57	A
THR	415	E	SER	56	A
GLY	416	E	TYR	52	A
GLY	416	E	SER	56	A
GLY	416	E	PHE	58	A
LYS	417	E	ASP	93	L
LYS	417	E	TYR	33	A
LYS	417	E	GLN	100	A
LYS	417	E	TYR	52	A
ASP	420	E	SER	56	A
ASP	420	E	PHE	58	A
ASP	420	E	TYR	52	A
TYR	421	E	TYR	52	A
TYR	421	E	SER	53	A
TYR	421	E	GLY	55	A
TYR	421	E	GLY	54	A

TYR	421	E	TYR	33	A
TYR	421	E	SER	56	A
TYR	449	E	ASN	31	L
TYR	453	E	VAL	101	A
TYR	453	E	PHE	32	L
ARG	454	E	TYR	33	A
LEU	455	E	GLN	100	A
LEU	455	E	VAL	101	A
LEU	455	E	TYR	33	A
LEU	455	E	LEU	99	A
PHE	456	E	TYR	33	A
PHE	456	E	TYR	102	A
PHE	456	E	LEU	99	A
PHE	456	E	ASP	98	A
PHE	456	E	GLN	100	A
PHE	456	E	ASN	32	A
ARG	457	E	SER	53	A
ARG	457	E	GLY	54	A
LYS	458	E	SER	31	A
LYS	458	E	GLY	54	A
LYS	458	E	SER	30	A
LYS	458	E	SER	53	A
SER	459	E	GLY	54	A
SER	459	E	SER	53	A
ASN	460	E	GLY	54	A
ASN	460	E	SER	56	A

ASN	460	E	GLY	55	A
ASN	460	E	SER	53	A
TYR	473	E	ASN	32	A
TYR	473	E	SER	30	A
TYR	473	E	SER	53	A
TYR	473	E	SER	31	A
GLN	474	E	SER	31	A
ALA	475	E	SER	31	A
ALA	475	E	GLY	26	A
ALA	475	E	ILE	27	A
ALA	475	E	THR	28	A
ALA	475	E	ASN	32	A
ALA	475	E	ARG	97	A
GLY	476	E	THR	28	A
GLY	476	E	ASN	32	A
GLY	476	E	SER	31	A
GLY	476	E	GLY	26	A
GLY	476	E	ILE	27	A
SER	477	E	GLY	26	A
SER	477	E	THR	28	A
GLU	484	E	TYR	102	A
PHE	486	E	GLY	26	A
PHE	486	E	ASP	105	A
PHE	486	E	VAL	106	A
PHE	486	E	ARG	97	A
PHE	486	E	ILE	27	A

PHE	486	E	VAL	2	A
ASN	487	E	GLY	26	A
ASN	487	E	LEU	99	A
ASN	487	E	ILE	27	A
ASN	487	E	ASP	105	A
ASN	487	E	THR	28	A
ASN	487	E	ASN	32	A
ASN	487	E	ARG	97	A
TYR	489	E	ARG	97	A
TYR	489	E	TYR	102	A
TYR	489	E	LEU	99	A
TYR	489	E	ASN	32	A
TYR	489	E	ASP	105	A
PHE	490	E	TYR	102	A
GLN	493	E	VAL	101	A
GLN	493	E	TYR	102	A
SER	494	E	PHE	32	L
TYR	495	E	PHE	32	L
TYR	495	E	SER	30	L
GLY	496	E	PHE	32	L
GLY	496	E	SER	30	L
PHE	497	E	SER	30	L
GLN	498	E	SER	30	L
GLN	498	E	GLY	68	L
GLN	498	E	SER	67	L
GLN	498	E	ASN	31	L

THR	500	E	SER	67	L
THR	500	E	THR	69	L
THR	500	E	ILE	29	L
THR	500	E	GLN	27	L
THR	500	E	GLY	28	L
THR	500	E	GLY	68	L
ASN	501	E	GLY	68	L
ASN	501	E	ILE	29	L
ASN	501	E	SER	30	L
ASN	501	E	GLY	28	L
GLY	502	E	GLN	27	L
GLY	502	E	GLY	28	L
GLY	502	E	ILE	29	L
VAL	503	E	GLN	27	L
GLY	504	E	GLN	27	L
TYR	505	E	HIS	90	L
TYR	505	E	ILE	2	L
TYR	505	E	ASN	92	L
TYR	505	E	GLN	27	L
TYR	505	E	GLY	28	L
TYR	505	E	ILE	29	L

*The total number of interfacial contacts is 121 which includes 2 charged-charged contacts; 10 charged-polar contacts; 17 charged-nonpolar contacts; 16 polar-polar contacts; 44 polar-nonpolar contacts; 32 nonpolar-nonpolar contacts.
