# Supplementary material for: Multiscale Modeling and Dynamic Mutational Profiling of Binding Energetics and Immune Escape for Class I Antibodies with SARS-CoV-2 Spike Protein: Dissecting Mechanisms of High Resistance to Viral Escape Against Emerging Variants
Source: Viruses. 2025 Jul 23;17(8):1029. doi: 10.3390/v17081029 (PMC12390076; doi:10.3390/v17081029)
Supplement: Supplementary file 1 [file viruses-17-01029-s001.zip › viruses-3717688-supplementary/SUPPLEMENTARY MATERIALS/Table S7.pdf]

Table S7. The list of the intermolecular contacts in the structure of the P5S-2B10 complex with RBD (pdb id 7XSC).

RBD Residue	RBD Residue Number	RBD Chain	Ab Residue	AB Residue Number	AB Chain
ARG	403	E	ASN	93	B
ARG	403	E	ASP	92	B
ASP	405	E	GLN	27	B
ASP	405	E	ASN	93	B
THR	415	E	THR	57	A
THR	415	E	SER	56	A
THR	415	E	PHE	58	A
GLY	416	E	TYR	52	A
GLY	416	E	PHE	58	A
GLY	416	E	SER	56	A
LYS	417	E	ASP	92	B
LYS	417	E	TYR	33	A
LYS	417	E	TYR	52	A
LYS	417	E	TYR	100	A
ASP	420	E	PHE	58	A
ASP	420	E	TYR	52	A
ASP	420	E	SER	56	A
TYR	421	E	SER	56	A
TYR	421	E	SER	53	A
TYR	421	E	TYR	33	A
TYR	421	E	TYR	52	A
TYR	421	E	GLY	54	A

TYR	421	E	GLY	55	A
TYR	453	E	ASP	92	B
TYR	453	E	PHE	32	B
LEU	455	E	SER	53	A
LEU	455	E	TYR	33	A
LEU	455	E	TYR	100	A
PHE	456	E	SER	53	A
PHE	456	E	TYR	33	A
PHE	456	E	SER	31	A
PHE	456	E	TYR	100	A
PHE	456	E	ASN	32	A
PHE	456	E	GLY	101	A
ARG	457	E	SER	53	A
ARG	457	E	GLY	54	A
LYS	458	E	ARG	71	A
LYS	458	E	SER	31	A
LYS	458	E	SER	53	A
LYS	458	E	SER	30	A
LYS	458	E	GLY	54	A
LYS	458	E	GLY	55	A
SER	459	E	GLY	54	A
SER	459	E	SER	53	A
ASN	460	E	SER	56	A
ASN	460	E	GLY	54	A
ASN	460	E	GLY	55	A
TYR	473	E	ASN	32	A

TYR	473	E	SER	31	A
TYR	473	E	SER	53	A
TYR	473	E	SER	30	A
GLN	474	E	SER	31	A
ALA	475	E	PHE	27	A
ALA	475	E	ARG	97	A
ALA	475	E	THR	28	A
ALA	475	E	ASN	32	A
ALA	475	E	SER	31	A
GLY	476	E	ASN	32	A
GLY	476	E	GLY	26	A
GLY	476	E	PHE	27	A
GLY	476	E	THR	28	A
SER	477	E	THR	28	A
SER	477	E	PHE	27	A
GLY	485	E	ASP	102	A
PHE	486	E	VAL	2	A
PHE	486	E	GLY	26	A
PHE	486	E	ASP	102	A
PHE	486	E	ARG	97	A
ASN	487	E	ASN	32	A
ASN	487	E	THR	28	A
ASN	487	E	GLY	26	A
ASN	487	E	ASP	102	A
ASN	487	E	PHE	27	A
ASN	487	E	ARG	97	A

CYS	488	E	ASP	102	A
TYR	489	E	ASN	32	A
TYR	489	E	ASP	102	A
TYR	489	E	ARG	97	A
TYR	489	E	GLY	101	A
GLY	496	E	ARG	30	B
GLN	498	E	ARG	30	B
THR	500	E	ARG	30	B
THR	500	E	ASP	28	B
ASN	501	E	ARG	30	B
ASN	501	E	ASP	28	B
GLY	502	E	ARG	30	B
GLY	502	E	ASP	28	B
TYR	505	E	ASN	93	B
TYR	505	E	PHE	32	B
TYR	505	E	ILE	29	B
TYR	505	E	ARG	30	B
TYR	505	E	ASP	28	B
TYR	505	E	ASP	92	B

*The total number of interfacial contacts is 103 which includes 3 charged-charged contacts; 15 charged-polar contacts; 22 charged-nonpolar contacts; 8 polar-polar contacts; 25 polar-nonpolar contacts; 20 nonpolar-nonpolar contacts.
